# A novel endoscopic classification for craniopharyngioma based on its origin

**DOI:** 10.1038/s41598-018-28282-4

**Published:** 2018-07-05

**Authors:** Bin Tang, Shen Hao Xie, Li Min Xiao, Guan Lin Huang, Zhi Gang Wang, Le Yang, Xuan Yong Yang, Shan Xu, Ye Yuan Chen, Yu Qiang Ji, Er Ming Zeng, Tao Hong

**Affiliations:** 1 Department of Neurosurgery, the First Affiliated Hospital of Nanchang University, No.17 Yongwai Street, Nanchang, Jiangxi Province 330006 People’s Republic of China; 2 Department of Pathology, the First Affiliated Hospital of Nanchang University, Nanchang, People’s Republic of China; 3 Department of Radiology, the First Affiliated Hospital of Nanchang University, Nanchang, People’s Republic of China

## Abstract

Endoscopic endonasal approach for craniopharyngioma (CP) resection provides a wide view and direct observation of hypothalamus and origin of tumor. Under endoscopy, 92 CPs were classified into 2 types: Peripheral and Central, according to its relation to pituitary stalk. Peripheral type was further divided into 3 subtypes: Hypothalamic stalk, Suprasellar stalk and Intrasellar stalk CP, according to the different origin site along hypothalamus-pituitary axis. Peripheral type arisen from the stalk but expanded and grown laterally in an exophytic pattern, accounting for 71.7% of all CPs, preservation rate of stalk was higher (76.0%). Central type grew within and along pituitary stalk and located strictly in the midline. The pituitary stalk was hardly preserved (only15.4%). Hypothalamic stalk CPs (n = 36, 54.6%) developed from the junction of hypothalamus and stalk, hypothalamus damage was found in all of this subtype after surgery. Suprasellar stalk CPs (n = 14, 21.2%) originated from the lower portion of stalk and displaced hypothalamus upward rather than infiltrated it. Intrasellar stalk CPs (n = 16, 24.2%) arose from the subdiaphragma portion of the stalk, with less hypothalamus damage. Recoginzing the origin of CP is helpful to understand its growth pattern and relation to hypothalamus, which is critical in planning the most appropriate surgical approach and degree of excision.

## Introduction

Craniopharyngioma (CP) is a benign epithelial tumor deriving from remnants of Rathke’s cleft, which degenerated along the hypothalamus-pituitary axis during embryonic period^1–3^. It is widely considered to be one of the most challenging intracranial tumor^4,5^. In some way, the dilemma of a radical removal of CP lies to hypothalamus and infundibular-stalk preservation against tumor recurrence^6–8^.

Understanding the pathological features of CP, including its origin, growth pattern and topographical relationship with surrounding vital structures, is critical in planning the most appropriate surgical approach and degree of excision^6,9–12^. At present, knowledge about the pathological changes of this entity has largely come from the studies of radiological images, observations during operations and autopsies^13,14^. However, image studies only provide information about the size, location and relationship with surrounding structures of CP, but seldom able to identify the origin site of tumors, the degree of adhesion and involvement of hypothalamus. Although observation during the operation can obtain more direct and clear morphological and topographic characteristics of lesion, direct visualization of hypothalamus and determination of its origin in each case is difficult, even impossible in conventional open approach due to the covers from the various location of optic chiasm, carotid artery or anterior communicating artery complex^15^.

In contrast, endoscopic endonasal approach provides an in-line access as well as superior visualization and illumination, leading us to definitely identify the pituitary stalk, hypothalamus and the origin of CP in nearly each case without any blind corner^16–26^. Realizing the exact place where CP is derived from along the hypothalamus-pituitary axis is fundamental to determine the definite relationship of tumor with hypothalamus, which is critical for safely resecting CP^4,27,28^. Although there have been a large number of reports on classification of CP^9,23,27,29–31^, one based on origin site of tumor is still lacking. We hypothesized that: 1) lesions derived from the different part of the hypothalamus-pituitary axis would have different growth pattern and different relation to surrounding structure, particularly the hypothalamus; 2) the origin site of the tumor would be the place where the infiltration or invasion of tumor to adjacent tissue occurs, but the other surface of the capsule would be the place where adherence with the surrounding structures occurs if happened. In other words, involvement of hypothalamus should basically include two types of relationships, that is the invasion (infiltration) and adherence (non infiltration). What type of relation to hypothalamus determines the surgical strategy, radical or planed limited resection.

Given the advantage of endoscopic observation, we retrospectively analyzed the radiological images, intraoperative findings and histological examinations of 92 consecutive cases which histologically confirmed CPs surgically treated through endoscopic endonasal approach. We then proposed a new classification based on different origin segment at the pituitary stalk and demonstrated the completely different involvement fashion of hypothalamus (e.g. infiltration or adhesion) in each subtype of CPs.

## Methods

A total of 129 patients with histopathologically confirmed CPs between December 2012 and June 2017 were identified, 91 patients who underwent purely endoscopic endonasal approach for tumor removal were included in this study, one patient with combined endoscopic endonasal and transcranial approaches was also included, 37 patients with open approach were excluded. The origin site of tumor, the relations of tumor to hypothalamus and pituitary stalk as well as clinical presentation, imaging studies, endocrinological studies, ophthalmological examinations, neuropsychological function, histopathological examination, and surgical complications of all 92 patients who underwent endoscopic endonasal approach for CPs were retrospectively reviewed through the medical records and intraoperative videos. Hospital review board approval was obtained for all aspects of this study. Informed consent had been approved by all patients included in this study.

### Endoscopic classification of CPs based on the stalk and the origin site

**Figure 1:**
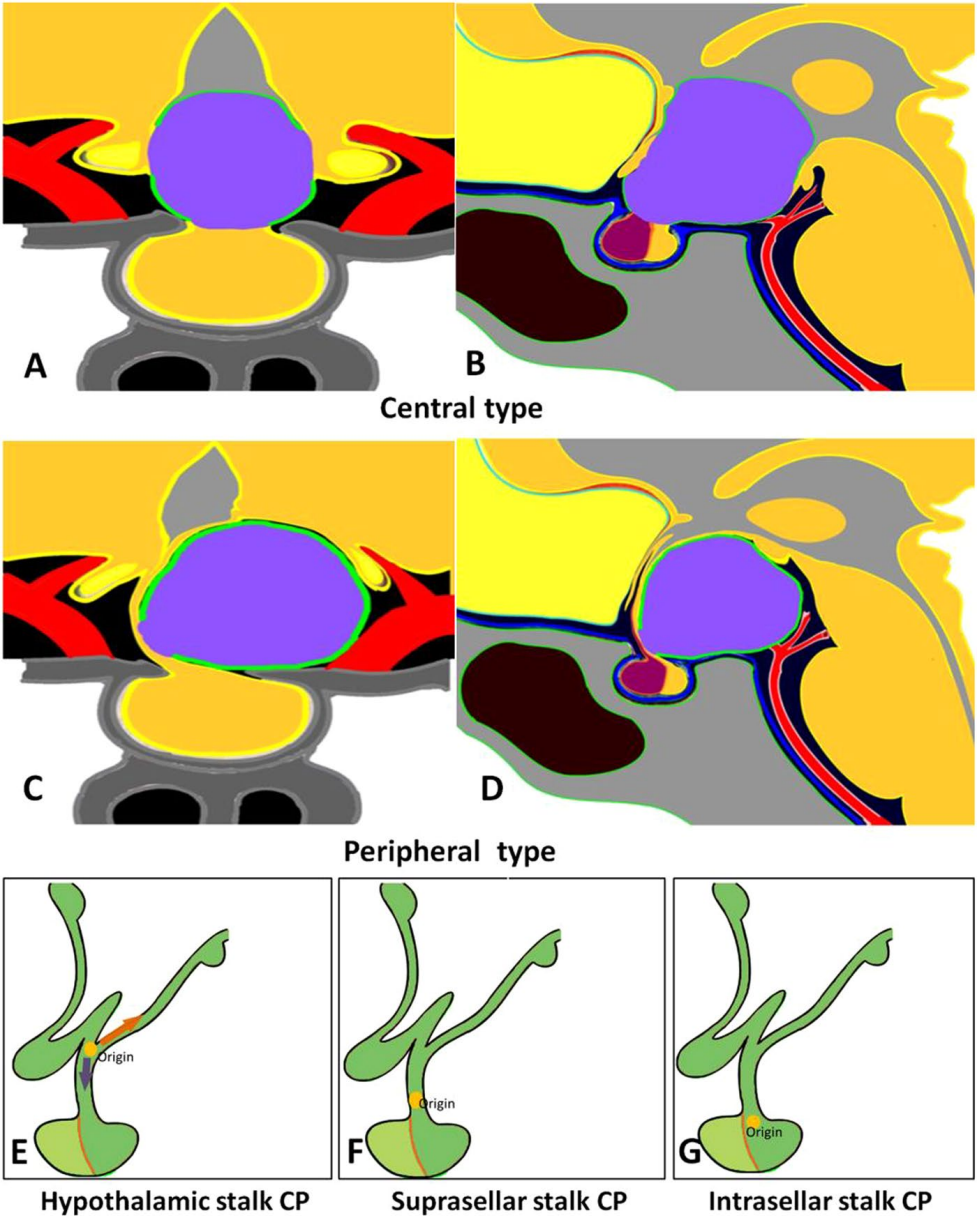
Schematic diagrams of the endoscopic subclassification of CPs. (**A,B**) Central type CP grows within and along the stalk and no pedicle or definite origin site can be identified, tumor is always located strictly in the midline. (**C,D**) Peripheral type CP arises from the stalk but expands and grows laterally in an exophytic pattern, the residual stalk is usually displaced to circumferential surface of the tumor. (**E**,**F** and **G**) Different origin sites of 3 subtypes of Peripheral type CPs along the pituitary stalk. (**E**) Hypothalamic stalk CP develops at the junction of the hypothalamus and the stalk, which usually extends up to the hypothalamus (brown arrow) and/or down to the up portion of stalk (black arrow), and usually invaded into the third ventricle. (**F**) Suprasellar stalk CP derives from suprasellar segment, usually low portion, of the stalk, and commonly locates extraventriclely. (**G**) Intrasellar stalk CP originates from the part of stalk under diaphragma, which is also known as intrasellar CP. Yellow discs in schematic diagrams indicate the origin site of the tumor.

**Figure 2:**
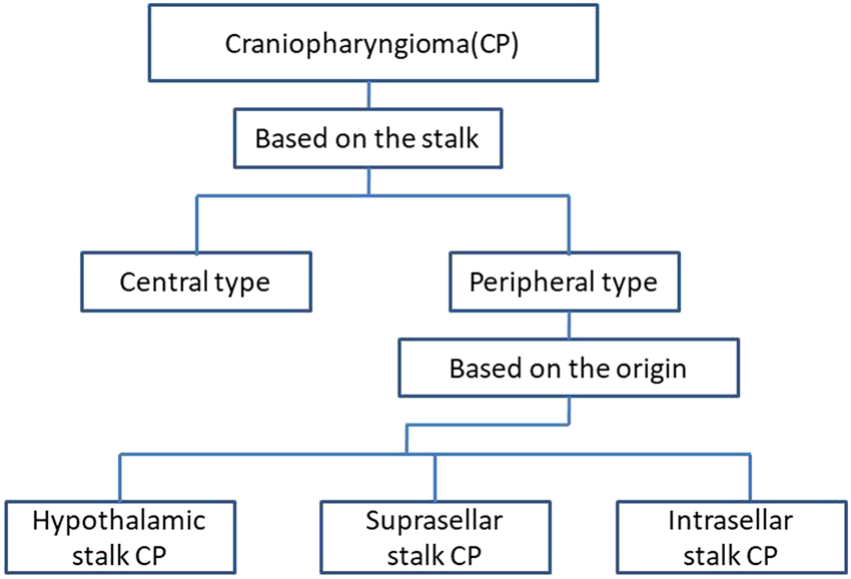
Scheme of the endoscopic classification of CPs based on the relation with stalk and the origin of tumor.

Based on the relationship between tumor and pituitary stalk, and origin site of the tumor, we proposed a new classification of CPs as follows (Fig. 1 and Fig. 2).

**Figure 3:**
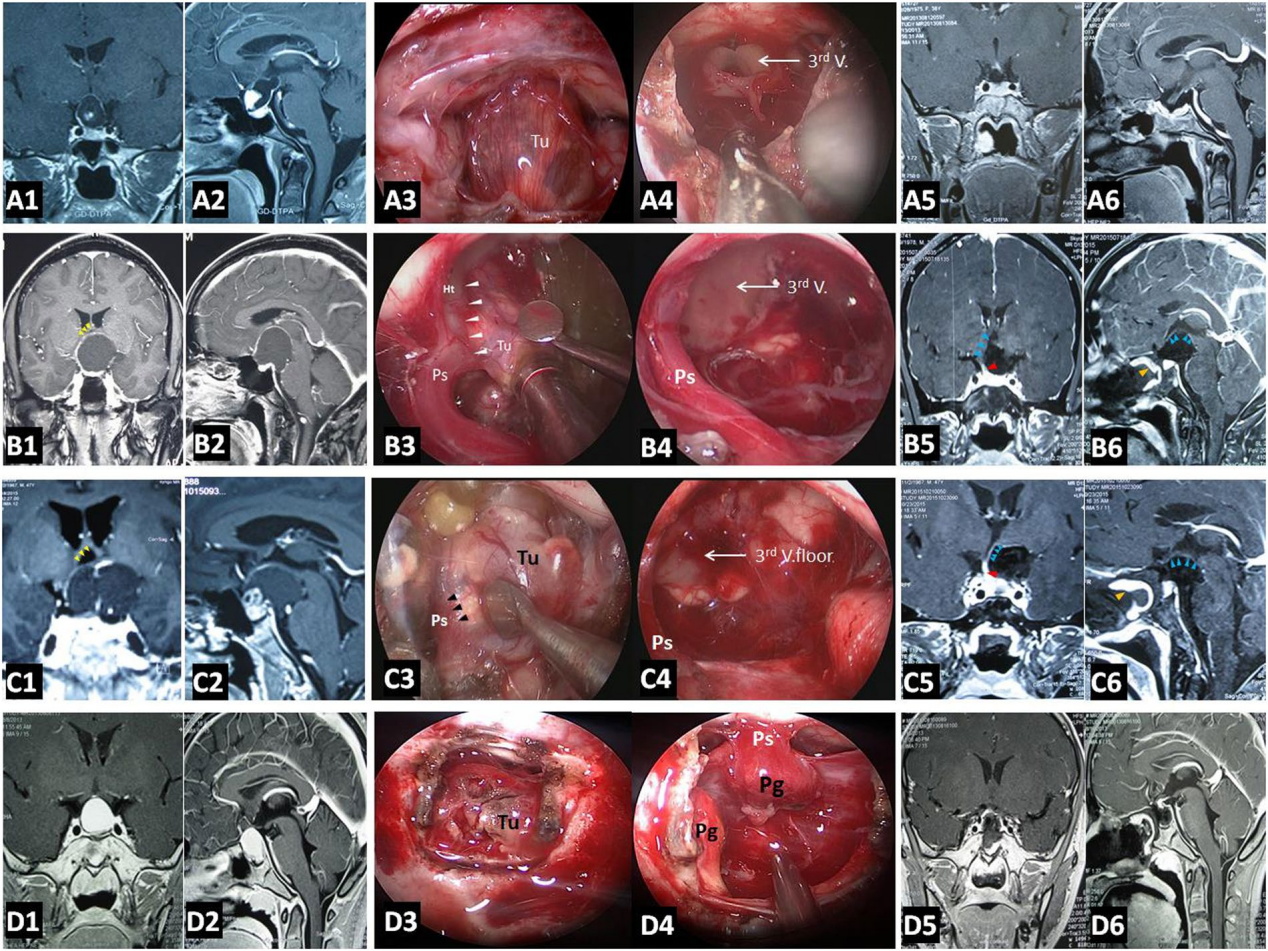
Pre- and post-operative images and intraoperative findings of each subtype of CPs via endoscopic endonasal approach. (**A1-6**) A case of Central type CP. (**A1-2**) Coronal and sagittal postcontrast T1-weighted MR images showing a Central type CP which is located in the midline. (**A3-4**) Intraoperative photographs showing that tumor grew within and along the stalk, and the characteristic structure of stalk could be clearly identified on the surface of tumor (**A3**). Bilateral hypothalamus damage limited to tuber cinereum area could be seen after tumor removal (**A4**). (**A5-6**) Coronal and sagittal postcontrast T1-weighted MR images obtained after GTR. The pituitary gland was preserved and intact, but the stalk was not preserved. (**B1-6**) A case of Hypothalamic stalk CP. (**B1-2**) Coronal and sagittal postcontrast T1-weighted MR images showing a suprasellar CP located slightly toward to the left side and grows into the third ventricle, with the anterior third ventricle shifted to the right side (yellow arrowheads in B1). (**B3-4**) Intraoperative photographs showing that tumor originated from the junction of the hypothalamus and the stalk (white arrowheads in B3), the stalk was pushed to the right side. After tumor removal, a defect at the left side of the third ventricle floor could be found, and the remnant of stalk was pushed to the right side and connected to the right side of the third ventricle floor, with a relative normal hypothalamus at the right side (B4). (**B5-6**) Coronal and sagittal postcontrast T1-weighted MR images showing total tumor removal achieved. The stalk was preserved and pushed to the right side (red arrowhead in B5), the right hypothalamus was intact (blue arrowheads in B5-6). Note the vascularized nasoseptal flap at the posterior aspect of the sphenoid sinus on the sagittal image (orange arrowhead in B6). (**C1-6**) A case of Suprasellar stalk CP. (**C1-2**) Coronal and sagittal postcontrast T1-weighted MR images showing a suprasellar CP located toward to the left side, with the anterior third ventricle shifted to the right side (yellow arrowheads in C1). (**C3-4**) Intraoperative photographs showing that tumor derived from suprasellar segment, low portion of the stalk (black arrowheads in C3). After tumor removal, the third ventricle floor was intact, with no hypothalamus damage, and the remanent of stalk was pushed to the right side (C4). (**C5-6**) Coronal and sagittal postcontrast T1-weighted MR images showing total tumor removal achieved. The stalk was preserved and pushed to the right side (red arrowhead in C5), and the hypothalamus was intact (blue arrowheads in C5-6). Note the vascularized nasoseptal flap at the posterior aspect of the sphenoid sinus on the sagittal image (orange arrowhead in C6). (**D1-6**) A case of Intrasellar stalk CP. (**D1-2**) Coronal and sagittal postcontrast T1-weighted MR images showing a typical intrasellar CP. (**D3-4**) Intraoperative photographs showing that the solid tumor located in the sellar region. Residual pituitary gland and stalk can be seen after tumor removal (D4). (**D5-6**) Coronal and sagittal postcontrast T1-weighted MR images showing total tumor removal achieved. Ht, hypothalamus; Tu, tumor; Pg, pituitary gland; Ps, pituitary stalk. 3rd V., the third ventricle.

For resection of a suprasellar CP via endoscopic endonasal approach, after opening dura mate and arachnoid membrane of suprasellar cistern, the first step was to find pituitary stalk. According the relationship between tumor and pituitary stalk, CP could be divided into 2 types. In one type, tumor grew within and along the stalk and no pedicle or definite origin site could be identified. We named it as Central type (Fig. 1A,B, Fig. 3A1-6), corresponding to Kassam’s transfundibular type^23^. In another type, tumor arose from the stalk but expanded and grew laterally in an exophytic pattern, the residual stalk was usually displaced to circumferential surface of the tumor, either positioned toward one side (right or left), or in front or back of the tumor. We named this growth pattern as Peripheral type (Fig. 1C and D), in which the pre and retro infundibular types of Kassam’s classification^23^ were included.

Next, after the position of pituitary stalk had been located, we then explored the origin site of the tumor along the pituitary stalk. In Central type, no definite origin site could be identified due to its transinfundibular growth pattern. But in Peripheral type, the base or pedicle of tumor was obvious, therefore, its origin could be determined. Although CPs could be developed anywhere from the junction of hypothalamus and stalk (top of the stalk), to the intrasellar segment (bottom of the stalk), generally they often occurred on one of three points (junction of hypothalamus and pituitary stalk, low portion of suprasellar stalk and intrasellar stalk) as we observed under endoscopy. The tumor from each site of origin demonstrated remarkably different pattern of relationship to hypothalamus or the third ventricle floor, which determined the extent of tumor resection. Based upon this characteristic, we further classified Peripheral type CP into three subtypes: Hypothalamic stalk CP, Suprasellar stalk CP and Intrasellar stalk CP. Hypothalamic stalk CP developed at the junction of the hypothalamus and stalk, which usually extended up to hypothalamus and/or down to the up portion of stalk, and usually invaded into the third ventricle (Fig. 1E, Fig. 3B1-6). Suprasellar stalk CP derived from suprasellar segment, usually low portion, of the stalk, and commonly located extraventriclely (Fig. 1F, Fig. 3C1-6). Intrasellar stalk CP originated from the part of stalk under diaphragma, which was also known as intrasellar CP (Fig. 1G, Fig. 3D1-6).

### Neuroradiological Evaluation

All patients underwent MR image, CT before and after surgery. Preoperatively, MR image was used to evaluate the tumor volume, consistency and location. Tumor volume was approximated by a modified ellipsoid volume, that is, (A × B × C) × π/6, A, B, and C represents the maximum diameters of CP in each of the 3 dimensions. The extent of intratumoral calcification was determined on CT scans. Anatomical features of the cerebral vessels were evaluated on preoperative angiograms.

To assess the extent of tumor resection, neuroradiologists independently reviewed the results of the preoperative and postoperative MR images and CT scans. Resection classifications were determined as follows: Gross-total resection (GTR) was defined as no residual enhanced lesion or residual calcification. In residual tumors, the degree of resection to be subtotal (STR) when ≥80% of the tumor was resected, and partial (PR) when <80% of the tumor was removed.

### Endocrinological evaluation

Serum levels of free triiodothyronine, free thyroxine, thyroid-stimulating hormone, luteinizing hormone, follicle-stimulating hormone, prolactin, growth hormone, cortisol, and adrenocorticotropic hormone were evaluated before and after surgery.

Pituitary deficiency was defined as the loss of function in single or multiple hormonal axis. Partial hypopituitarism was defined as two axis hormone deficiencies, and panhypopituitarism was defined as three or more axes hormone deficiencies. Hyperprolactinemia was encountered at times and attributes to stalk effect. Diabetes insipidus (DI) was diagnosed before and after surgery based on the sodium level and the presence of hypotonic polyuria.

### Visual Assessment

Visual acuity and computerized visual field examinations were performed in all patients preoperatively. During postoperative follow-up, these examinations were performed at 6 months and 18 months after surgery; Examinations were further repeated when clinically appropriate.

### Neuropsychological Function and Performance Status

Neuropsychological function was evaluated pre and postoperatively. Cognitive function, sleep quality, functional performance status were evaluated using the Montreal Cognitive Assessment (MOCA), Stanford Sleepiness Scale (SSS), Karnofsky Performance Scale (KPS) respectively.

### Intraoperative observation

With intraoperative observation and reviewing the operation videos, the following items were carefully evaluated: the location of the pituitary stalk, the origin site of the tumor, the base or pedicle of the tumor (tumor base located only in the origin site without any extension was defined narrow, before resection of tumor, narrow tumor base could be seen completely under endoscopy; while tumor base located not only in the origin site but also with extension was defined broad, before resection of tumor, only inferior part of broad tumor base could be seen), growth pattern of the tumor, the relations of tumor to hypothalamus and pituitary stalk, extent of hypothalamus damage and the greater calcification plaque (D > 1 cm).

### Histopathological examination

Histopathological examinations of the typical lesions were completed by two experienced pathologists independently. The following items were carefully evaluated: the pathological type of tumor, the origin site of the tumor, the relation of tumor to hypothalamus (infiltration or adherence) and pituitary stalk.

### Statistical Analysis

Data were collected using Microsoft Excel 2010 (Microsoft Corp.) and analyzed using SPSS 21.0. A p value < 0.05 was considered to be statistical significant.

## Results

### Patient Population and Clinical presentation

This group consisted of 74 adults (42 men and 32 women, mean age 44.8 years, range 20–70 years) and 18 children (11 boys and 7 girls, mean age 13.2 years, range 9–17years). 15 patients had previously been surgically treated before the admission. In all these 15 patients, 12 patients had previously undergone surgery via a transcranial approach, followed by gamma knife therapy in 3 patients among them, 2 patients had undergone transsphenoid microsurgery, and another 1 patient had previously undergone gamma knife surgery and stereotactic cyst aspiration in other institution.

The most common clinical manifestation was pituitary dysfunction, which occurred in 64 patients (69.6%), followed by visual impairment (65.2%), headache (60.9%), DI (17.4%), overweight (17.4%) and memory deterioration (10.9%). In adult patients, visual impairment occurred in 52 (70.3%) of them, pituitary dysfunction in 67.6%, headache in 62.2%, DI and overweight in 13.5%. In pediatric patients, pituitary dysfunction occurred in 14 (77.8%) patients, headache in 55.6%, visual deficit in 44.4% and overweight in 33.3% (Table 1).

**Table 1:** Clinical manifestation of 92 patients with craniopharyngiomas.

Groups	Adults (n[%])	Children (n[%])	Total (n[%])
No. of patients	74	18	92
Visual impairment	52 (70.3)	8 (44.4)	60 (65.2)
Headache	46 (62.2)	10 (55.6)	56 (60.9)
Pituitary dysfunction	50 (67.6)	14 (77.8)	64 (69.6)
Hypogonadism	30 (40.5)	12 (66.7)	42 (45.7)
Hypoadrenalism	4 (5.4)	0 (0.0)	4 (4.3)
GH deficit	0 (0.0)	10 (55.6)	10 (10.9)
Hypothyroidism	30 (40.5)	8 (44.4)	38 (41.3)
Hyperprolactinemia	12 (16.2)	2 (11.1)	14 (15.2)
DI	10 (13.5)	6 (33.3)	16 (17.4)
Overweight	10 (13.5)	6 (33.3)	16 (17.4)
Memory deterioration	10 (13.5)	0 (0.0)	10 (10.9)
Nausea/vomiting	2 (2.7)	2 (11.1)	4 (4.3)
CN palsy	0 (0.0)	2 (11.1)	2 (2.2)
Gait disturbance	2 (2.7)	0 (0.0)	2 (2.2)
Mental changes	4 (5.4)	0 (0.0)	4 (4.3)

GH, growth hormone; DI, diabetes insipidus; CN, cranial nerve.

### Tumor classification

Of the 92 tumors examined in this study, 26 (28.3%) were classified as Central type, and 66 (71.7%) as Peripheral type. According to the different origin site along hypothalamus-pituitary axis, Peripheral type could be further divided into 3 subtypes: 36 (39.1%) were classified as Hypothalamic stalk CPs, 14 (15.2%) as Suprasellar stalk CPs, 16 (17.4%) as Intrasellar stalk CPs. The number of men and women and the distribution of age in each subtype of CPs were showed in Table 2.

**Table 2:** Tumor classification of 92 CPs under endoscopy.

Groups	Central type	Peripheral type		
		Hypothalamic stalk CP	Suprasellar stalk CP	Intrasellar stalk CP
No. of patients (%)	26 (28.3)	36 (39.1)	14 (15.2)	16 (17.4)
Sex (M/F) (n)	14/12	19/17	12/2	9/7
Age (Adults/Children) (n)	24/2	30/6	14/0	6/10

### Neuroradiological Findings

The average tumor volume in all patients was 17.84 cm^3^ (range 1.51–66.70 cm^3^), and the maximum tumor diameter varied from 1.6 to 6.3 cm. In adult patients, the average tumor volume was 15.19 cm^3^ (range1.51–66.70 cm^3^) and the maximum tumor diameter ranged from 2.0 to 6.3 cm. In pediatric patients, the average tumor volume was 28.52 cm^3^ (range 1.76–43.30 cm^3^), and the maximum tumor diameter ranged from 1.6 to 6.2 cm. In regard to consistency, mixed (solid-cystic) tumors were found in 72 (78.3%) patients, calcifications of different degrees were present in 29 (31.5%) cases. In this series, 76 pure supradiaphragmatic lesions (82.6%) were found both in adulthood (91.9%) and childhood (44.4%), and 16 intrasellar lesions were found in adulthood (8.1%) and childhood (55.6%) (Table 3).

**Table 3:** Craniopharyngioma characteristics based on preoperative imaging findings.

**Characteristic**	**Adults (n[%])**	**Children (n[%])**	**Total (n[%])**
**No. of patients**	74	18	92
**Maximum diameter**			
≥3 cm	48 (64.9)	14 (77.8)	62 (67.4)
<3 cm	26 (35.1)	4 (22.2)	30 (32.6)
**Consistency**			
Cystic	12 (16.2)	2 (11.1)	14 (15.2)
Solid	4 (5.4)	2 (11.1)	6 (6.5)
Mixed	58 (78.4)	14 (77.8)	72 (78.3)
**Calcification**			
Yes	16 (21.6)	13 (72.2)	29 (31.5)
No	58 (78.4)	5 (27.8)	63 (68.5)
**Location**			
Suprasellarsupradiaphragmatic	68 (91.9)	8 (44.4)	76 (82.6)
Intrasellar	6 (8.1)	10 (55.6)	16 (17.4)

In preoperative MR image, 54 (81.8%) Peripheral type CPs with the maximum diameter larger than 3 cm, while only 8 (30.8%) Central type CPs larger than 3 cm. Large calcifications (D>1 cm) were present in 12 (18.2%) Peripheral type CPs and 6 (23.1%) Central type CPs, respectively. All 26 Central type CPs located strictly in the midline and without any shift of the third ventricle. Except 16 Intrasellar CPs, 32 (48.5%)Peripheral type CPs located more or less toward to one side, and 31 (47.0%) Peripheral type CPs with the shift of the third ventricle to one side in the coronal MR image (Table 4).

**Table 4:** Imaging characteristics of Peripheral type and Central type CPs.

Feature	Peripheral type (n[%])	Central type (n[%])
**Case number**	66	26
**Maximum diameter**		
≥3 cm	54 (81.8)	8 (30.8)
<3 cm	12 (18.2)	18 (69.2)
**Calcification** (**D > 1** **cm**)		
Yes	12 (18.2)	6 (23.1)
No	54 (81.8)	20 (76.9)
**Location in coronal MR image (except Intrasellar CPs)**		
Slightly toward to one side	32 (48.5)	0 (0.0)
Midline	20 (30.3)	26 (100.0)
**3**^**rd**^ **Ven**. **in coronal MR image (except Intrasellar CPs)**		
Shifted to one side	31 (47.0)	0 (0.0)
Midline	19 (28.8)	26 (100.0)

### Intraoprative findings

**Figure 4:**
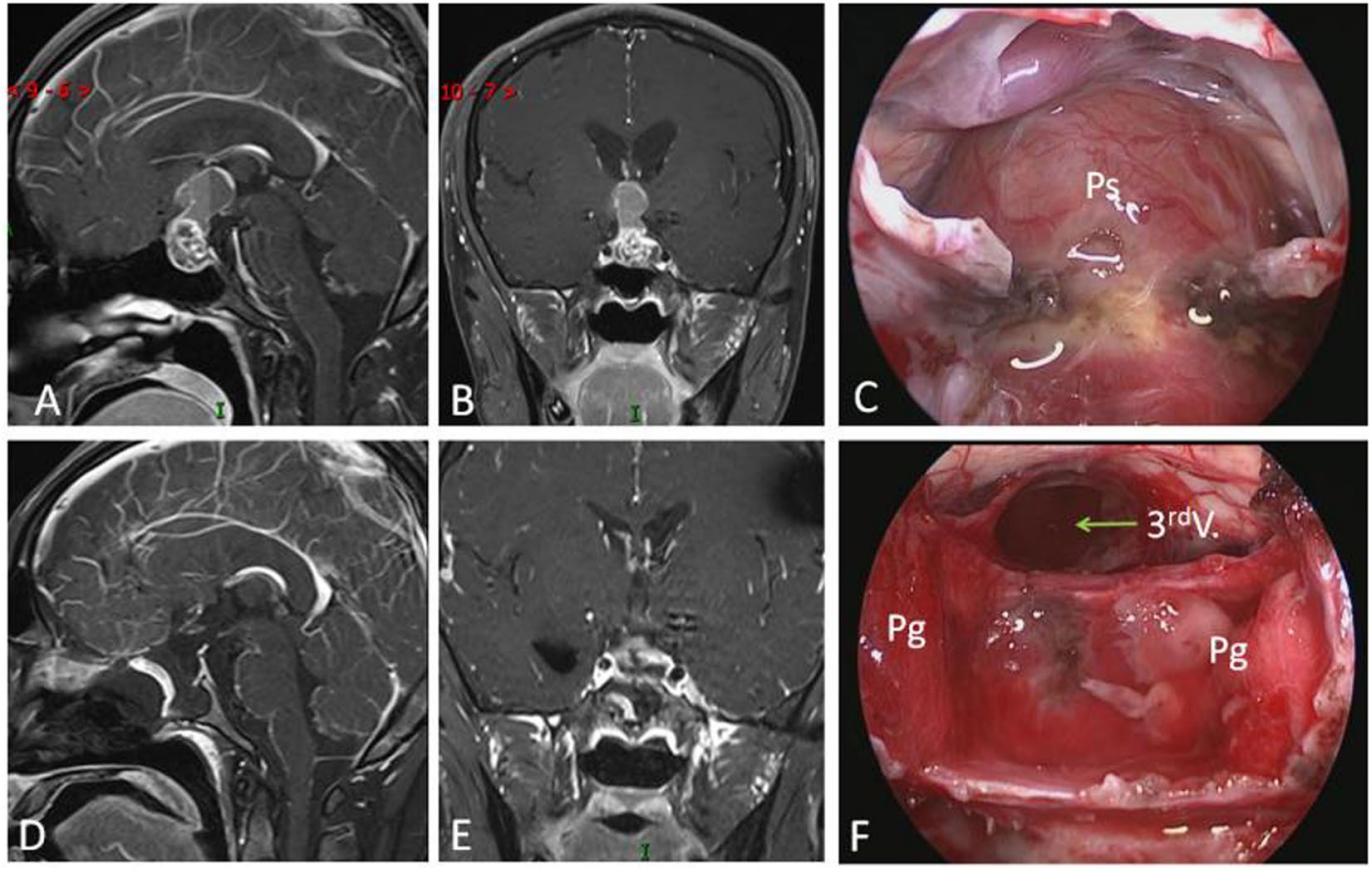
Example of a Central type Cp grows longer in vertical direction. (**A** and **B**) Sagittal (A) and Coronal (B) postcontrast T1-weighted MR images showing that tumor grows up to the third ventricle and down to the intrasellar region through pitiutary stalk. (**C** and **F**) Intraoperative photographs showing that tumor grew within and along the stalk and down to the intrasellar region (C). The third ventricle floor was destroyed, residual pituitary gland pushed and compressed by the tumor could be seen after tumor removal (F). (**D** and **E**) Sagittal (D) and Coronal (E) postcontrast T1-weighted MR images obtained after GTR demonstrating extensive, safe resection of the tumor. 3^rd^ V., the third ventricle; Pg, pituitary gland.

Peripheral type was the most common CP, accounting for 71.7% of all CPs. The pituitary stalk was usually displaced to the side of tumor and its preservation rate was higher (76.0%) after tumor resection. In contrast, Central type grew within and along pituitary stalk and located strictly in the midline, with the hypothalamus damage. The tumor was frequently smaller, but some cases might grow longer in vertical direction, up to the third ventricle and/or down to the intrasellar region (Fig. 4). The pituitary stalk encompassed the tumor surface and was hardly preserved (only 15.4%).

Hypothalamic stalk CPs always originated from the hypothalamus and upper portion of the stalk, with the hypothalamus damage. Among 36 Hypothalamic stalk CPs, 30 (83.3%) tumors with the maximum diameter larger than 3 cm, large calcifications (D > 1 cm) were present in 10 (27.8%) cases. The tumor grew often in vertical direction, with one part of tumor in suprasellar cistern, another part in the third ventricle. In admantinomatous type, the ventricle part was often cystic, while the cisternal part was often solid or solid-cystic, where plaque calcification could be found. The tumor base or “pedicle” of this type of Cps could be relatively broad or narrow. Among 36 Hypothalamic stalk CPs, we found that 28 (77.8%) CPs had broad base, and tended to extending more to hypothalamus rather than the stalk. The low part of stalk usually appears normal or slightly enlarged (Fig. 3B3-4). Hypothalamus damage was found in all CPs of this type. Pituitary stalks were preserved in 26 (72.2%) patients with Hypothalamic stalk CPs.

Suprasellar stalk CPs originated frequently from the lower portion of stalk, and rarely infiltrated hypothalamus but could push it upward. Among 14 Suprasellar stalk CPs, only 2 cases (14.3%) broken through and into the third ventricle. This subtype of CP tended to grow into a giant tumor in a roughly horizontal level along the cisterns or skull base, with extension to the parasellar region, prepontine and posterior fossa, anterior fossa and sylvian cistern. In our series, 10 (71.4%) Suprasellar stalk CPs presented such growth pattern. Tumor base of this subtype of CPs was always narrow (71.4%), compared to Hypothalamic stalk CPs (22.2% with narrow pedicle, 77.8% with broad pedicle). Pituitary stalks were preserved in 12 (85.7%) Suprasellar stalk CPs.

Intrasellar stalk CPs originated from the subdiaphragmatic portion of stalk, could extend to suprasellar space, cavernous sinus and sphenoid sinus, and we supposed the direction of growth depended on the thickness of the sellar diaphragma, inner wall of cavernous sinus and floor of sellar turcica. In this study, 6 (37.5%) Intrasellar stalk CPs grew into giant tumors in the vertical direction, pushed the hypothalamus upward, but with less hypothalamus damage (Table 5).

**Table 5:** Intraoperative findings in 92 patients with craniopharyngiomas.

**Groups**	**Central type (n[%])**	**Peripheral type**			**Total (n[%])**
		**Hypothalamic stalk CP (n[%])**	**Suprasellar stalk CP (n[%])**	**Intrasellar Stalk CP (n[%])**	
**Case number**	26	36	14	16	92
**Maximum diameter**					
≥3 cm	8 (30.8)	30 (83.3)	10 (71.4)	14 (87.5)	62 (67.4)
<3 cm	18 (69.2%)	6 (16.7)	4 (28.6)	2 (12.5)	30 (32.6)
**Calcification** (**D > 1 cm)**					
Yes	6 (23.1)	10 (27.8)	1 (7.1)	1 (6.3)	18 (19.6)
No	20 (76.9)	26 (72.2)	13 (92.9)	15 (93.7)	74 (80.4)
**Relations w/ hypothalamus**					
None	/	0 (0.0)	0 (0.0)	10 (62.5)	10 (10.9)
Push	/	0 (0.0)	12 (85.7)	6 (37.5)	18 (19.6)
Invade	26 (100%)	36 (100.0)	2 (14.3)	0 (0.0)	64 (69.5)
**Tumor base**					
Broad	/	28 (77.8)	4 (28.6)	/	32 (34.8)
Narrow	/	8 (22.2)	10 (71.4)	/	18 (19.6)
**3** ^**rd**^ **Ven. part properties**					
Cyst	20 (76.9)	28 (77.8)	2 (14.3)	/	50 (54.3)
Solid	6 (23.1)	8 (22.2)	0 (0.0)	/	14 (15.2)
**Preservation of stalk**	4 (15.4)	26 (72.2)	12 (85.7)	/	42 (45.7)

### Extent of Tumor Resection

Among the 92 cases, GTR was achieved in 78 (84.8%) patients, STR was achieved in 12 (13.0%) patients, and PR was achieved in only 2 patients (2.2%). Among the 62 patients with giant tumors (maximum diameter ≥3 cm), GTR was achieved in 50 (80.6%) patients, STR was achieved in 10 (16.1%) patients, and PR was achieved in only 2 patients (3.2%). Among the 29 cases with calcification, GTR was achieved in 23 (79.3%) patients, STR was achieved in 4 (13.8%) patients, and PR was achieved in only 2 patients (6.9%).

In all 26 patients with Central type CPs, GTR was achieved in 24 (92.3%) patients, STR in 2 (7.7%) patients, and no patient had PR. In all 66 patients with Peripheral type CPs, GTR was achieved in 54 (81.8%) patients, STR in 10 (15.2%) and PR in 2 (3.0%) patients.

Among the 36 patients with Hypothalamic stalk CPs, GTR was achieved in 30 (83.3%) patients, STR in 4 (11.1%) patients and PR in 2 (5.6%) patients. In the 14 patients with Suprasellar stalk CPs, GTR was achieved in 10 (71.4%) patients, STR in 4 (28.6%) patients and no patient had PR. In the 16 patients with Intrasellar stalk CPs, GTR was achieved in 14 (87.2%) patients, STR in 2 (12.5%) patients and no patient had PR (Table 6).

**Table 6:** Extent of tumor removal with regard to tumor features in 92 patients with craniopharyngiomas.

**Groups**	**Total (n)**	**GTR (n[%])**	**STR (n[%])**	**PR (n[%])**
**Case number**	92	78 (84.8)	12 (13.0)	2 (2.2)
**Maximum diameter**				
≥3 cm	62	50 (80.6)	10 (16.1)	2 (3.2)
<3 cm	30	28 (93.3)	2 (6.7)	0 (0.0)
**Consistency**				
Cystic	14	10 (71.4)	4 (28.6)	0 (0.0)
Solid	6	4 (66.7)	2 (33.3)	0 (0.0)
Mixed	72	64 (88.9)	6 (8.3)	2 (2.8)
**Calcification**				
Yes	29	23 (79.3)	4 (13.8)	2 (6.9)
No	63	55 (87.3)	8 (12.7)	0 (0.0)
**Classification**				
Central type	26	24 (92.3)	2 (7.7)	0 (0.0)
Peripheral type	66	54 (81.8)	10 (15.2)	2 (3.0)
Hypothalamic stalk CP	36	30 (83.3)	4 (11.1)	2 (5.6)
Suprasellar stalk CP	14	10 (71.4)	4 (28.6)	0 (0.0)
Intrasellar stalk CP	16	14 (87.2)	2 (12.5)	0 (0.0)

### Histopathological examination

The surgical specimens of the origin and non-origin capsule of CPs were pathologically studied respectively in 8 cases, including 4 cases with Hypothalamic CPs, 3 cases with Suprasellar stalk CPs and one case of Intrasellar stalk CP.

**Figure 5:**
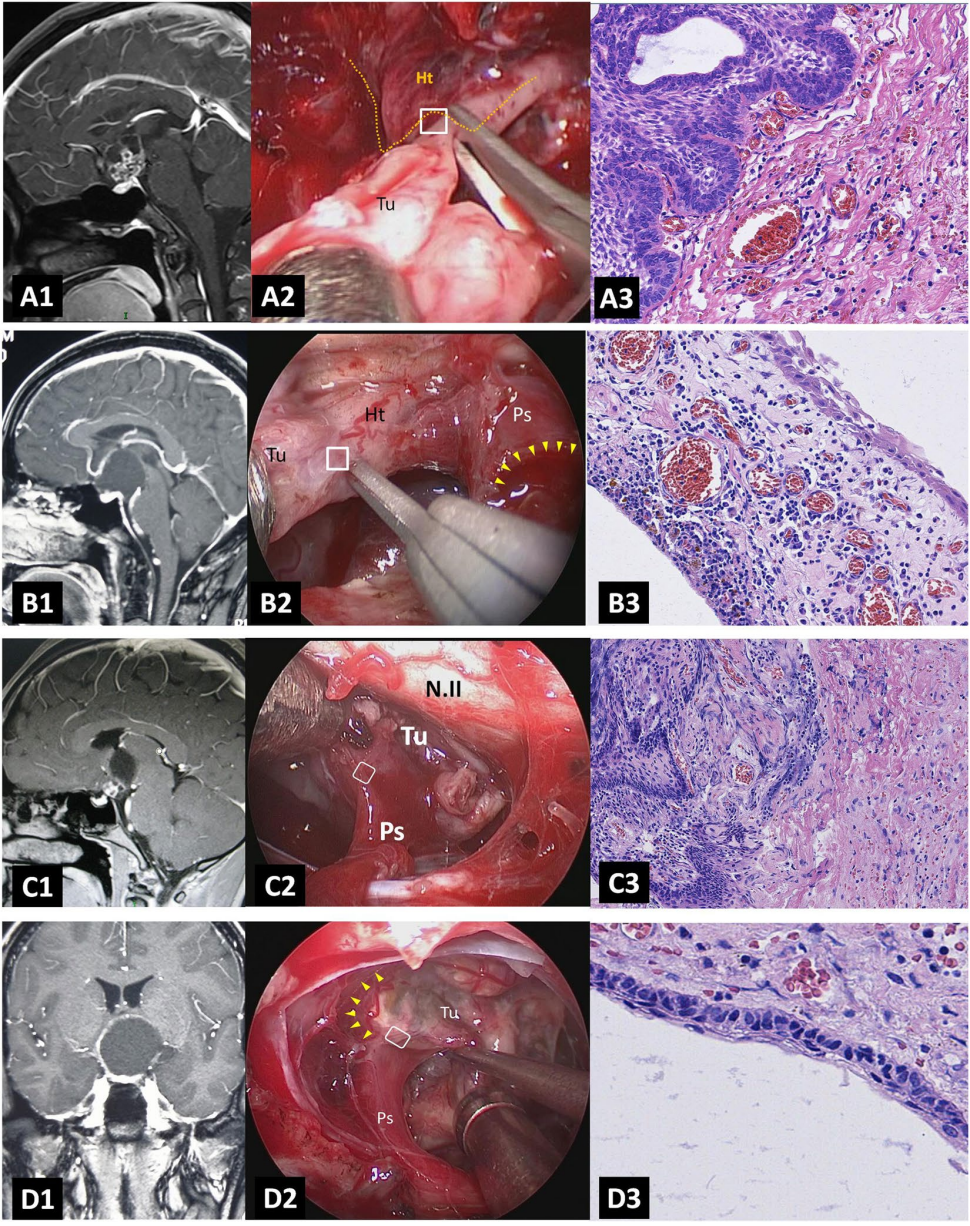
Histopathological examination of the origin and non-origin capsule of CPs. (**A1-3**) Relationship between origin site of Hypothalamic stalk CP and hypothalamus. (**A1**) Sagittal postcontrast T1-weighted MR image showing a typical Hypothalamic stalk CP which grows up to the third ventricle. (**A2**) Intraoperative photograph showing that the specimen was obtained from the junction of hypothalamus and stalk (white box), yellow dashed line indicates the board of hypothalamus. (**A3**) Histopathological examination (×200) of tissue in the white box (B) showing that tumor invades the hypothalamus with interdigitation or finger-like infiltration. (**B1-3**) Relationship between non-origin capsule of Suprasellar stalk CP and hypothalamus. (**B1**) Sagittal postcontrast T1-weighted MR image showing a typical Suprasellar stalk CP which is located extraventriclely and pushes hypothalamus upward. (**B2**) Intraoperative photograph showing that the specimen was obtained from the non-origin capsule of tumor attached to hypothalamus (white box). Yellow arrowheads indict the origin site of tumor which located in the lower part of stalk. (**B3**) Histopathological examination (×200) of tissue in the white box (B) showing that the non-origin capsule of tumor is full of tumor cells, without any hypothalamus tissues. (**C1-3**) Relationship between origin site of Hypothalamic stalk CP and pituitary stalk. (**C1**) Sagittal postcontrast T1-weighted MR image showing a Hypothalamic stalk CP which grows up to the third ventricle. (**C2**) Intraoperative photograph showing that the specimen was obtained from the inferior part of the tumor base attached to the top of stalk (white box). (**C3**) Histopathological examination (×200) of tissue in the white box (B) showing the invasion of the tumor tissue into pituitary stalk. (**D1-3**) Relationship between non-origin capsule of Hypothalamic stalk CP and pituitary stalk. (**D1**) Coronal postcontrast T1-weighted MR image showing a Hypothalamic stalk CP which pushes the anterior third ventricle to the right side. (**D2**) Intraoperative photograph showing that the specimen was obtained from the non-origin capsule of tumor closed to the stalk (white box). yellow arrowheads indicate the origin site of tumor. (**D3**) Histopathological examination (×200) of tissue in the white box (B) showing the clear boundary between tumor and pituitary stalk which is without any tumor cells invasion. N.II, optic nerve; Ht, hypothalamus; Tu, tumor. Ps, pituitary stalk.

Typical interdigitation or finger-like infiltration protruding into the hypothalamus was observed in the origin tissue specimens in Hypothalamic stalk CPs (Fig. 5A1-3), whereas in the specimens of tight adherence area of hypothalamus in Suprasellar stalk CPs, this typical histopathological feature was not observed (Fig. 5B1-3).

In Hypothalamic stalk CPs and Suprasellar CPs, the stalk where the tumor originated was full of tumor cells infiltration interspersed with the remnants of stalk structure (Fig. 5C1-3). Although extensively adhering to surrounding structure such as optic chiasm and basal ganglion, the non-origin capsule showed a relative smooth and clear, or irregular interface without any interdigitated infiltration (Fig. 5D1-3).

### Endocrinological Outcome

In all 26 patients with Central type CPs, endocrinological results showed that 6 cases had normal anterior pituitary function, but all got worse after surgery. 10 cases reporting preoperative partial hypopituitarism got worse after surgery. 2 cases with preoperative panhypopituitarism were still unchanged postoperatively. 4 patients had DI preoperatively, none of them resolved after surgery. There were 10 new cases with DI postoperatively, 6 patients had transient DI, whereas another 4 patients had long-time DI.

In all 36 patients with Hypothalamic stalk CPs, 12 cases had normal anterior pituitary function preoperatively, but all got worse after surgery. 2 cases reporting preoperative partial hypopituitarism got worse after surgery, another 7 cases unchanged. 4 cases with preoperative panhypopituitarism were still unchanged postoperatively. 10 patients had DI preoperatively, only 2 patients resolved after surgery, another 8 patients were still unchanged. There were 32 new cases with DI postoperatively, 18 patients had transient DI, whereas another 14 patients had long-time DI.

In all 14 patients with Suprasellar stalk CPs, 5 cases had normal anterior pituitary function preoperatively, but all got worse after surgery. 2 cases reporting preoperative partial hypopituitarism were still unchanged after surgery. 2 cases with preoperative panhypopituitarism were still unchanged postoperatively either. 2 patients had DI preoperatively both resolved after surgery. There were 9 new cases with DI postoperatively, 3 patients had transient DI, whereas another 6 patients had long-time DI.

In all 16 patients with Intrasellar stalk CPs, 5 cases had normal anterior pituitary function preoperatively, but all got worse after surgery. 2 cases reporting preoperative partial hypopituitarism were still unchanged after surgery. There was no one presented preoperative panhypopituitarism and diabetes insipidus. There were 5 new cases with DI postoperatively, 3 patients had transient DI, whereas another 2 patients had long-time DI (Table 7).

**Table 7:** Preoperative and postoperative statuses in 92 patients with craniopharyngiomas.

**Groups**	**Central type (n)**	**Peripheral type**			**Total (n[%])**
		**Hypothalamic stalk CP (n)**	**Suprasellar stalk CP (n)**	**Intrasellar Stalk CP (n)**	
**Endocrinological status**					
Normal anterior pituitary function	6	12	5	5	28
Unchanged	0	0	0	0	0 (0.0)
Worsening	6	12	5	5	28 (100.0)
Partial hypopituitarism	10	9	2	2	23
Unchanged	0	7	2	2	11 (47.8)
Improved	0	0	0	0	0 (0.0)
Worsening	10	2	0	0	12 (52.2)
Panhypopituitarism	2	4	2	0	8
Unchanged	2	4	2	0	8 (100.0)
Improved	0	0	0	0	0 (0.0)
Worsening	0	0	0	0	0 (0.0)
Preop DI	4	10	2	0	16
Unchanged	4	8	0	0	12 (75.0)
Resolved	0	2	2	0	4 (25.0)
New cases of DI	10	32	9	5	56
Transient DI	6	18	3	3	30 (53.6)
Long-time DI	4	14	6	2	26 (46.4)
**Visual function**					
Preop normal	14	10	0	8	32
Unchanged	13	9	0	8	30 (93.8)
Worsening	0	0	0	0	0 (0.0)
Preop visual impairment	12	26	14	8	60
Unchanged	4	4	6	4	18 (30.0)
Improved	8	22	8	4	42 (70.0)
Worsening	0	0	0	0	0 (0.0)
Normalized	6	18	6	2	32 (53.3)
**Obesity**					
Preop	0	2	0	0	2 (2.2)
Postop	2	6	2	0	10 (11.1)

### Visual Outcome

Prior to surgery, 32 patients had normal visual function, including 14 patients with Central type CPs, 10 patients with Hypothalamic stalk CPs and 8 patients with the Intrasellar stalk CPs. All these patients with unchanged visual function after surgery except 2 death cases.

Concerning visual impairments preoperatively, we found overall improvement of the visual defect in 42 (70.0%) of 60 patients, including 8 patients with Central type CPs, 22 patients with Hypothalamic stalk CPs, 8 patients with Suprasellar stalk CPs and 4 patients with Intrasellar stalk CPs. Normalization of the impairment was achieved in 32 (53.3%) patients, while remained unchanged in 18 (30.0%) patients. We did not found new onset of any postoperative visual deficiency (Table 7).

### Obesity

Prior to surgery, only 2 (2.2%) of the 92 patients were considered to be obese, both with Hypothalamic stalk CPs. During follow-up, 10 patients (11.1%) were obese, including 2 patients with Central type CPs, 6 patients with Hypothalamic stalk CPs and 2 patients with Suprasellar stalk CPs. None of the patients with the Intrasellar stalk CPs was obese either before the surgery or at the last follow-up. The mean time of follow-up in our study was 29.1 months (range 11–56 months) (Table 7).

### Cognitive function, sleep quality and quality of life

The cognitive function was measured with MOCA scale, the mean preoperative and 6 month postoperative MOCA scores for patients with Central type CPs were 24.7 ± 4.2 and 18.8 ± 8.1, respectively, there was a significant difference between them (P = 0.000). For patients with Hypothalamic stalk CPs were 24.9 ± 7.3 and 23.9 ± 7.2, respectively, there was a significant difference between them (P = 0.002). For patients with Suprasellar stalk CPs were 28.4 and 28.3, respectively, and for patients with Intrasellar stalk CPs were 27.5 ± 0.5 and 27.4 ± 1.2, respectively, there was no significant difference between them (P = 0.604, 0.806, respectively).

The quality of sleep was evaluated with SSS, the mean preoperative and 6 month postoperative SSS scores for patients with Central type CPs were 2.67 ± 0.78 and 3.25, respectively, there was a significant difference between them (P = 0.012). For patients with Hypothalamic stalk CPs were 3.18 ± 1.70 and 3.59 ± 1.70, respectively, there was no significant difference between them (P = 0.248). For patients with Suprasellar stalk CPs were 2.29 ± 0.49 and 2.14 ± 0.90, respectively, and for patients with Intrasellar stalk CPs were 2.00 ± 0.73 and 2.31 ± 0.95, respectively, there was no significant difference between them (P = 0.604, 0.055, respectively).

Quality of life was evaluated with KPS scale, the mean preoperative and 6 month postoperative KPS scores for patients with Central type CPs were 82.5 ± 9.7 and 72.5 ± 16.6, respectively, there was a significant difference between them (P = 0.011). For patients with Hypothalamic stalk CPs were 76.5 ± 23.7 and 78.8 ± 14.1, respectively, there was no significant difference between them (P = 0.496). For patients with Suprasellar stalk CPs were 90.0 ± 0.0 and 92.9 ± 4.9, respectively, and for patients with Intrasellar stalk CPs were 87.5 ± 4.5 and 88.7 ± 9.6, respectively, there was no significant difference between them (P = 0.172, 0.544, respectively) (Table 8).

**Table 8:** Preoperative and postoperative functional performance statuses in 92 patients with craniopharyngiomas. CI: confidence interval.

**Central type**				**Peripheral type**								
				**Hypothalamic stalk CP**			**Suprasellar stalk CP**			**Intrasellar stalk CP**		
	**Mean ± SD**	**r** (**CI95%)**	**P**	**Mean ± SD**	**r (CI95%)**	**P**	**Mean ± SD**	**r (CI95%)**	**P**	**Mean ± SD**	**r (CI95%)**	**P**
**MOCA**												
Preop	24.7 ± 4.2	5.9 (3.2 to 8.6)	0.000	24.9 ± 7.3	1.0 (0.4 to 1.6)	0.002	28.4 ± 0.5	0.1 (−0.5to 0.8)	0.604	27.5 ± 0.5	0.1 (−0.5 to 0.6)	0.806
Postop	18.8 ± 8.1			23.9 ± 7.2			28.3 ± 1.1			27.4 ± 1.2		
**SSS**												
Preop	2.67 ± 0.78	−0.58 (−0.19 to −1.01)	0.012	3.18 ± 1.70	−0.41 (−1.14 to 0.32)	0.248	2.29 ± 0.49	0.14 (−0.50 to 0.78)	0.604	2.00 ± 0.73	−0.31 (−0.63 to 0.01)	0.055
Postop	3.25 ± 0.62			3.59 ± 1.70			2.14 ± 0.90			2.31 ± 0.95		
**KPS**												
Preop	82.5 ± 9.7	10.0 (2.8 to 17.2)	0.011	76.5 ± 23.7	−2.4 (−9.5 to 4.8)	0.496	90.0 ± 0.0	−2.9 (−7.4 to 1.7)	0.172	87.5 ± 4.5	−1.25 (−5.55 to 3.05)	0.544
Postop	72.5 ± 16.6			78.8 ± 14.1			92.9 ± 4.9			88.7 ± 9.6		

### Surgical Complications

New hypopituitarism and permanent DI have already been described above in detail. CSF leakage was another common postoperative complication. Overall, CSF leakage occurred in 6.5% of patients, including 2 patients with Central type CPs, 2 patients with Hypothalamic stalk CPs and 2 patients with Suprasellar stalk CPs, and meningitis developed in these patients. All these patients were treated with reoperation, and 5 cases were saved.

Among other complications, demyelination, epilepsy, hydrocephalus were occurred with an incidence of 4.3%, 4.3% and 2.2% respectively. The postoperative mortality rate, defined as death within 30 days postoperatively, was 2.2% (2 of 92); One patient with an infundibularintraventricular partially cystic tumor with dense adhesion to the floor of the third ventricle died after 9 days of operation; The other patient died due to postoperative pneumonia. Only 2 Hypothalamic stalk CPs recurrent during the follow-up period (Table 9).

**Table 9:** Complications in 92 patients with craniopharyngiomas.

**Surgical complications**	**Central type (n)**	**Peripheral type**			**Total (n[%])**
		**Hypothalamic stalk CP (n)**	**Suprasellar stalk CP (n)**	**Intrasellar Stalk CP (n)**	
CSF leak	2	2	2	0	6 **(**6.5)
Meningitis	2	2	2	0	6 **(**6.5)
Epilepsy	1	3	0	0	4 **(**4.3)
Demyelination	0	4	0	0	4 **(**4.3)
Hydrocephalus	0	2	0	0	2 **(**2.2)
Pneumonia	2	0	0	0	2 **(**2.2)

## Discussion

An accurate definition of the topographical relationships between CPs and the surrounding neurovascular structures is essential prior to planning the most appropriate surgical approach and degree of excision, and predicting the postoperative morbidity and prognosis of the patients^28,32^. Based upon intraoperative findings and/or preoperative MR images, some authors have reported their classifications to topographically grade CPs according to their relation with diaphragm^31,33^, ventricle^9,30,34^, tumor extension^29^, stalk or infundibular^23^, arachnoid membrane^27^, optic nerve^35^ and all above^36^. However, at present there is no consensus on a standard reference classification^37^. The category based on the origin site is still lacking. Knowing the origin of CPs is helpful to understand their growth pattern and their relationship with surrounding structures, particularly with the hypothalamus. Although we have recognized that CPs can arise from anywhere along hypophyseal axis for long time, we are unable to directly visibly identify the exact tumor origin in each individual case, due to the cover of structures like optic nerve, chiasm and carotid artery, and the narrow operative view under microscope with transcranial approach. In contrast, endoscopic endonasal approach provides a direct and wider panorama of the space on which CPs originate from and grow, leading us to definitely identify the pituitary stalk, hypothalamus and the origin of CPs. Owing to the superior observation of endoscope, we developed a new classification based on the position of stalk and tumor origin. Each category demonstrated a distinct growth pattern and relation of tumor to hypothalamus.

### Endoscopic classification based on the stalk

With endoscopic endonasal approach, after opening of the dura and arachnoid membrane, the origin of suprasellar CP, as well as pituitary stalk and tumor topographical relations to surrounding structure can be clearly visualized. Based on the tumor position with stalk, Kassam *et al*.^23^ has classified CPs into preinfundibular, transinfundibular and retroinfundibular types. However, it is hard to use this classification scheme preoperatively because identifying the stalk is extremely difficult in pre-operative MR images, particularly in large and transinfundibular tumors. In addition, the stalks are more often displaced to left or right sides of the lesions, which hardly assign neither to pre- nor retro-infundibular type according to this classification scheme. Therefore, we modify this classification into 2 types: Peripheral type and Central type, which can be identified relatively easier in pre-operative MR image study, and the characteristics of both types are distinct.

Central type accounts for 28.3% of CPs. The tumor often has smaller volume and grows along and within the stalk axis. It is located strictly on the midline on the MR image. Some cases can grow longer in vertical direction, up to the third ventricle and down to the intrasellar region through pitiutary stalk. Bilateral hypothalamus damage is present after gross tumor resection, but usually limits to tuber cinereum area due to small size of tumor. The reason for small size in this type is probably due to the early and severe damage of the stalk. With aggressive resection, preserving the stalk is very difficult and not essential owing to its severe damage by lesion. Occasionally, relatively larger Central type CP can be seen, in which extensive damage of bilateral hypothalamus could happen, resulting in complete opening of the third ventricle without any residual hypothalamus if radical CP resection is performed.

**Figure 6:**
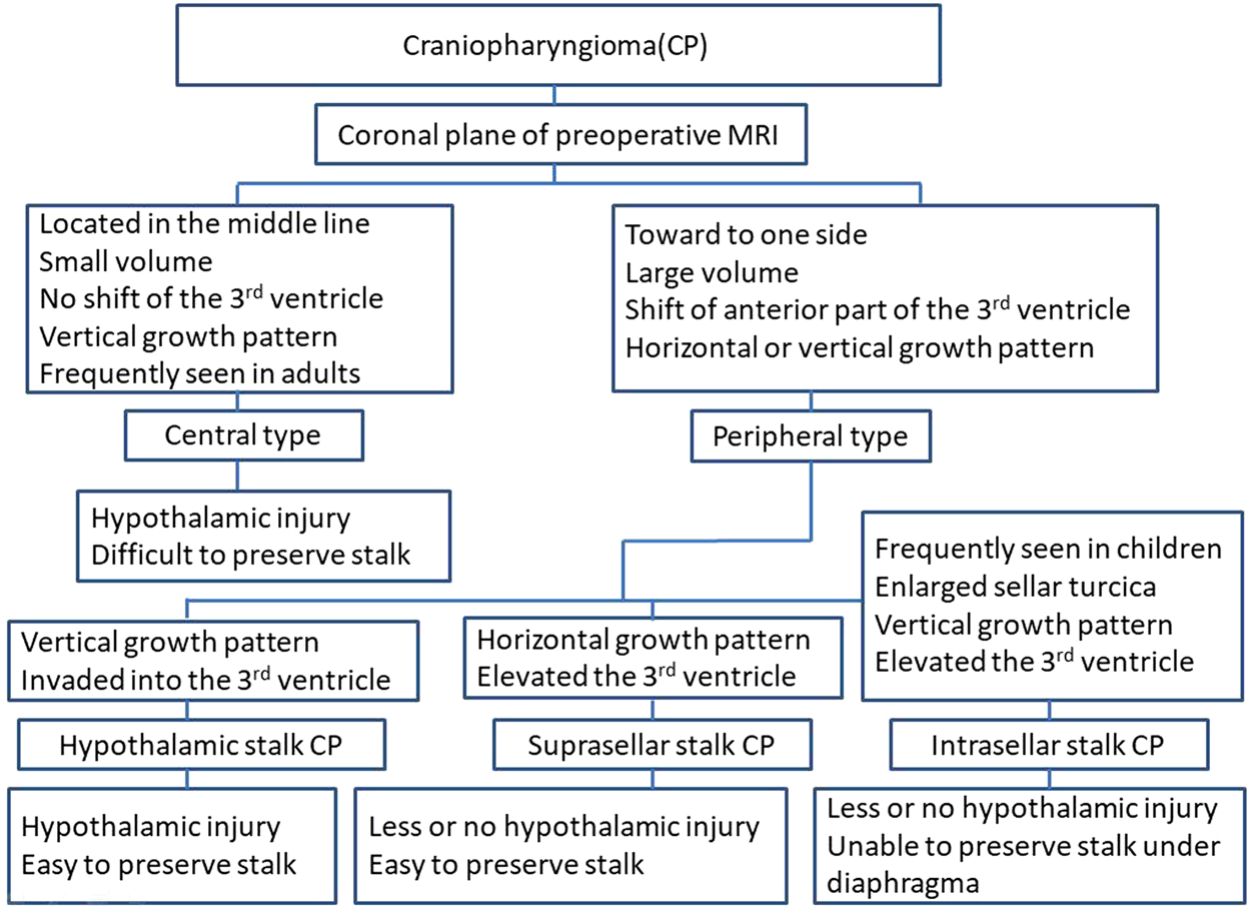
Illustration of the steps to diagnose the subtype of CP preoperatively and its corresponding degree of hypothalamus and stalk injury.

Peripheral type represents the most common category, accounts for 71.7% of all cases. Tumors develop from the hypothalamus-pituitary axis, but expand and grow slightly toward one side in an exophytic pattern. The stalk and residual hypothalamus are displaced to the periphery, more commonly to one side. Hypothalamus damage is present after gross tumor resection, but with some degree of residue depending upon the extent of hypothalamus involvement. In this type, the stalk preservation rate is higher, as compared to the Central type. In preoperative MR images, tumor grows not strictly symmetrically on the midline as Central type does, but slightly toward to one side (Fig. 3B1,C1). On the coronal MR image, the anterior third ventricle is slightly shifted to the other side, indicating the side of the stalk position (Fig. 2B1,C1). Therefore, the location of stalk can be preoperatively predicted from image study without a need to identify the stalk itself. In preoperative MR image study, the differential diagnosis between these two types is relatively easier. Tumor of Central type is usually smaller and located strictly in the midline, while mostly Peripheral type is larger and slightly toward to one side with the third ventricle shifted laterally (Fig. 6). The difference between the Central and Peripheral type is illustrated in Table 10.

**Table 10:** Characteristics of Central type and Peripheral type CPs.

**Feature**	**Central type**	**Peripheral type**
Case number (n[%])	26 **(**28.3%)	66 **(**71.7%)
Maximum diameter (≥3 cm)(n)	8	54
Location	Located midline	Slightly toward to one side
PS preservation rate (%)	15.4	76.0
Hypothalamic damage	Bilateral (100.0%)	Ipsilateral **(**83.3% in Hypothalamic stalk CPs)
3^rd^Ven in coronal MR image	Midline (100.0%)	Shifted to one side (62.0%)

### Endoscopic classification based on the origin site

Under endoscopic observation, tumor base or origin site can be clearly identified in Peripheral type. Based on the anatomic area and frequency of the tumor base location along the vertical hypothalamus-pituitary axis, Peripheral CP can be further classified into three categories: 1. Hypothalamic stalk CP, tumor originates from hypothalamus and upper portion of stalk; 2. Suprasellar stalk CP, tumor arises from the lower portion of suprasellar stalk; 3. Intrasellar stalk CP, tumor develops from the intrasellar portion of stalk.

### Hypothalamic stalk CP

**Figure 7:**
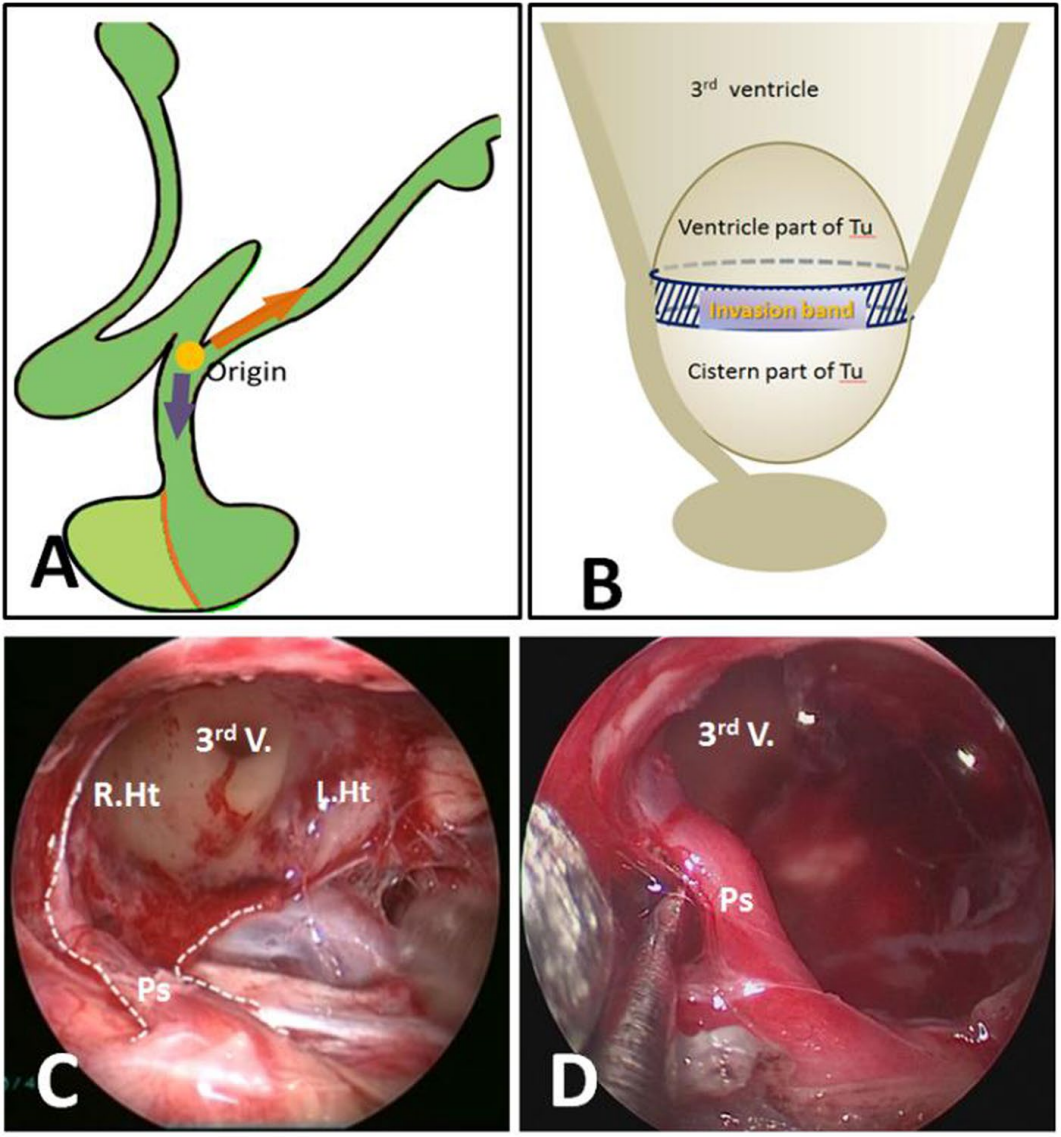
(**A** and **B**) Schematic drawing of Hypothalamic stalk CPs, which originate from the junction of hypothalamus and stalk, and can grow along the hypothalamus upward, or along the stalk downward, or both directions. The most common growth pattern is on both directions, and between the two parts, there is a circumferential band surrounded by tight adherence of the hypothalamic remnants (B). (**C** and **D**) Intraoperative photographs showing that a large defect at the left side of the third ventricle floor was observed after completely resection of a Hypothalamic stalk CP. The stalk was pushed to contralateral side and connected to the remnants of contralateral hypothalamus. Ps, pituitary stalk; 3^rd^ V., the third ventricle, L.Ht, left hypothalamus, R.Ht, right hypothalamus.

**Figure 8:**
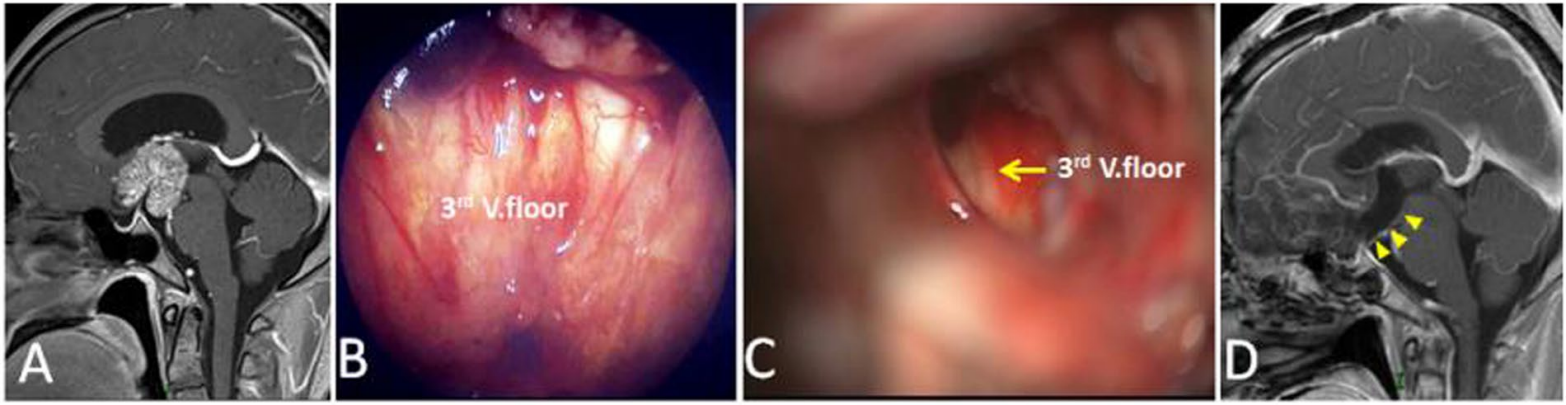
Example of a purely intraventricle Cp. (**A**) Sagittal postcontrast T1-weighted MR image showing a solid tumor only grows into the third ventricle. (**B** and **C**) Intraoperative photographs showing that the third ventricle floor was intact after tumor removal via the callosal-interforniceal approach. (**D**) Sagittal postcontrast T1-weighted MR images obtained after GTR. Yellow arrowheads indicate the intact the third ventricle floor. 3rd V., the third ventricle.

**Figure 9:**
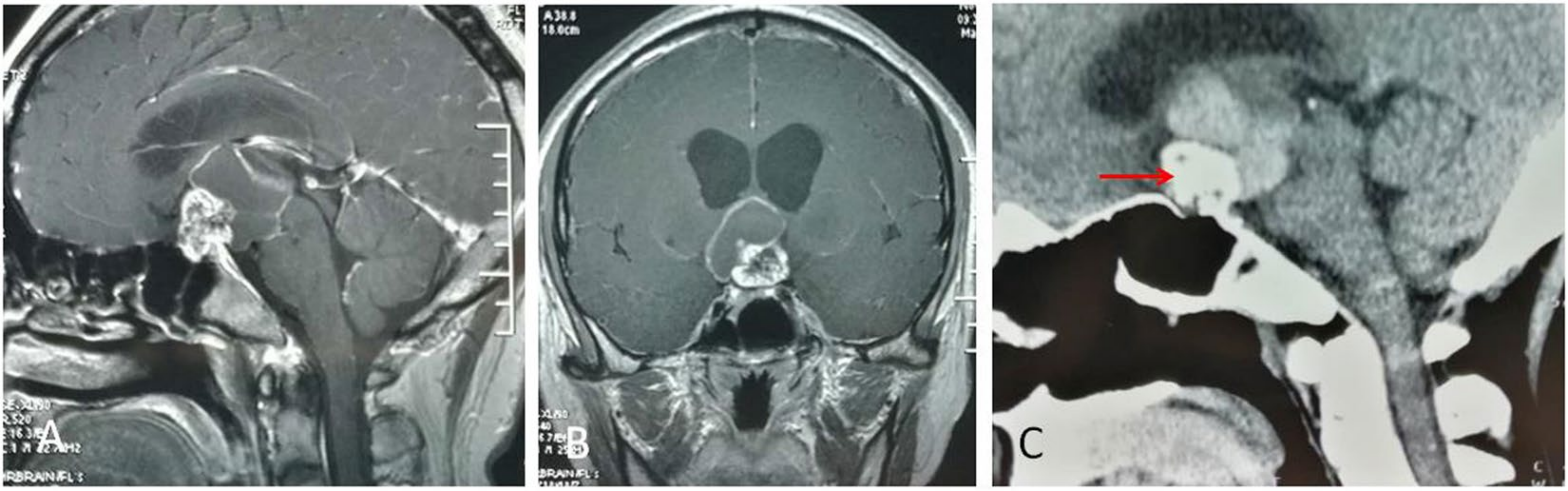
Example of a Hypothalamic stalk Cp (pathological confirmed as adamantinomatous Cp) grows in vertical direction. (**A** and **B**) Sagittal (A) and Coronal (B) postcontrast T1-weighted MR images showing that the upward growth tumo towards to the third ventricle is cystic, while the downward growth tumor towards to suprasellar cistern is solid. (**C**) The CT scan showing that plaque calcification can be found in the cisternal part (red arrow).

Hypothalamic stalk CPs represent the most common category, account for 40.5% adult CPs (Table 2) and one of the type that hypothalamus is invaded or the infiltrated by the tumor. CPs originate from the hypothalamus and upper portion of the stalk, i.e. the junction of hypothalamus and stalk, and can grow in vertical direction along the hypothalamus upward, or along the stalk downward, or both directions (Fig. 1E). The most common growth pattern is on both directions, with one part of tumor in suprasellar cistern, another part in the third ventricle. Between the two parts, there is a circumferential band surrounded by tight adherence of the hypothalamic remnants (Fig. 7B). This type of CP meets the criterion of infundibulo-tuberal CP or not strictly intraventricle CP, first described by Erdheim^38^ and recently reviewed by Pascual *et al*.^14^. If the tumor derives from hypothalamus and grows only in one direction into the third ventricle with an intact third ventricle floor, it meets the definition of “purely” intraventricle CP^33,34,39,40^ (Fig. 8). However, it occurs very rear, only account for only 2% in our series. Therefore, in our classification, purely intraventricle CP is an extreme variant of Hypothalamic stalk CP. On the contrary, tumor originated from the junction of hypothalamus and stalk also can grow only downward into the cistern, but not into the third ventricle. It is another extreme variant of Hypothalamic stalk CP, and also occurs very rare and we only had two cases with this subtype among all 92 patients. In this variant, the third ventricle is partly involved, but not penetrated by tumor and also kept intact. The origin area or tumor base or “pedicle” of Hypothalamic stalk CPs can either be broad or narrow, but usually broad, and tends to extends more to hypothalamus rather than the stalk. The low part of stalk usually appears normal or slightly enlarged. Interestingly, the base mostly limited to one side of hypothalamus, and a large defect at one side of the floor of the third ventricle is often observed after CP is completely removed (Fig. 7C). The stalk is often pushed to opposite side and connects to the remnants of opposite hypothalamus (Fig. 7D). In the adamantinomatous CP, the upward growth tumors within the ventricle are often cystic and without calcification, while the downward growth tumors within the suprasellar cistern are often solid or solid-cystic, where plaque calcification is often be found (Fig. 9).

### Suprasellar stalk CP

**Figure 10:**
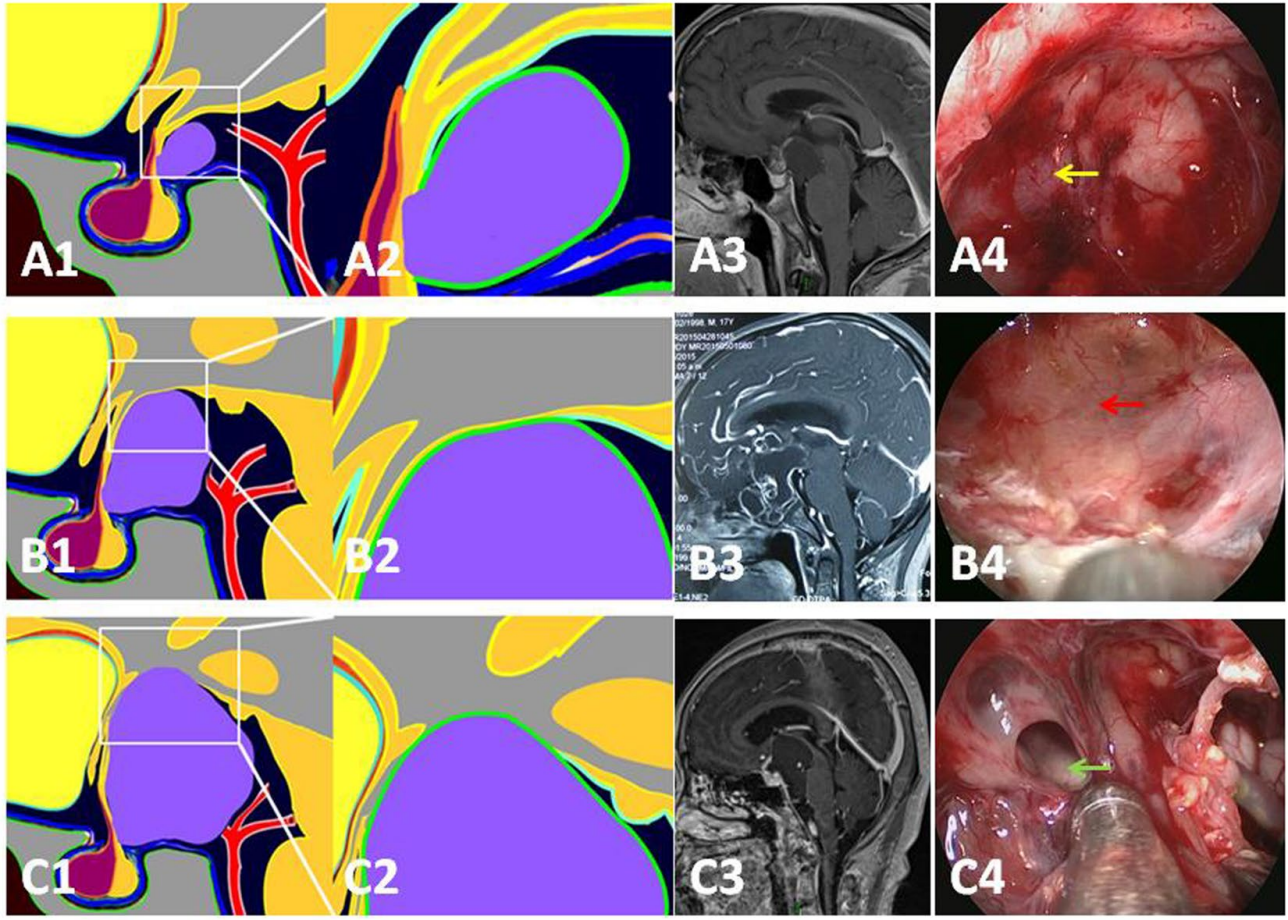
Three stages in the Suprasellar stalk CPs according to the relationship between tumor and hypothalamus. (**A1** and **A2**) Schematic drawing of a Suprasellar stalk CP in Grade 1: tumor contacts the hypothalamus but there exists a subaracnoid and pial membrane between them. (**A3**) The T1-GDPA image of a typical case in the sagittal plane of MR image. (**A4**) Intraoperative photographs showing that the third ventricle floor was thick and intact after tumor resection (yellow arrow). (**B1** and **B2**) Schematic drawing of a Suprasellar stalk CP in Grade 2: tumor obviously pushes the hypothalamus upward with no membrane between them. (**B3**) The T1-GDPA image of a typical case in the sagittal plane of MR image. (**B4**) Intraoperative photographs showing that the third ventricle floor was thin and intact after tumor resection (red arrow). (**C1** and **C2**) Schematic drawing of a Suprasellar stalk CP in Grade 3: tumor severely pushes the hypothalamus upward and breaks it into the third ventricle with tight adherence to the remnants of hypothalamus. (**C3**) The T1-GDPA image of a typical case in the sagittal plane of MR image. (**C4**) Intraoperative photographs showing that a defect of the third ventricle floor could be seen after tumor resection (blue arrow).

Suprasellar stalk CPs originate from the lower portion of stalk, account for 18.9% adult CPs (Table 2). The tumor rarely infiltrates hypothalamus but can push it upward. Based on the relationship between the tumor and hypothalamus, we grade it into three stages: Grade 1 (Fig. 10A1-4), the tumor contacts the hypothalamus but there exists a subaracnoid and pial membrane between them. It is easy to separate the tumor from hypothalamus with no postoperative hypothalamic response after tumor resection; Grade 2 (Fig. 10B1-4), the tumor obviously pushes the hypothalamus upward with no membrane between them. Separating the tumor from hypothalamus causes a slight postoperative hypothalamic response after tumor resection; Grade 3 (Fig. 10C1-4), the tumor severely pushes the hypothalamus upward and penetrates it into the third ventricle with tight adherence to the remnants of hypothalamus. Separating the tumor from hypothalamus causes a severe, but less than Hypothalamic stalk CPs, postoperative hypothalamic response after tumor resection. However, Grade 3 is very rear, probably because the tumor tends to extend in horizontal direction along the cisterns surrounding the stalk where the tumor derived from, compared to the vertical growth pattern of Hypothalamic stalk CP. More often than Hypothalamic stalk CPs, Suprasellar stalk CPs can grow into a giant tumor with extension to the parasellar region, prepontine and posterior fossa, anterior fossa and sylvian cistern, while Hypothalamus stalk CPs more often extend to the third ventricle and lateral ventricle. Furthermore, Suprasellar stalk CPs can grow downward into intrasellar fossa, either through pushing the diaphragm downward or along the stalk into the sellar fossa, which need to differentiate from Intrasellar stalk CPs. Considering the characteristics of this type of CP, correct option of surgical approaches is decisive, and translamina terminals or transventricle approach is forbidden in the case with Grade 1 and Grade 2, otherwise, the surgery would cause iatrogenic damage of the intact hypothalamus. Generally, the derangement of hypothalamic function is no (in Grade 1) or less (in Grade 2 and 3) compared to that of Hypothalamus CPs in the perioperative period and long term follow-up (Table 8).

### Intrasellar stalk CP

Intrasellar stalk CPs originate from the subdiaphragmatic portion of stalk and often be seen in children, about 55.6% in children CPs, and 8.1% in adult CPs (Table 2). The tumor can extend to suprasellar space, cavernous sinus and sphenoid sinus, and the direction of growth probably depends on the thickness of the sellardiaphragma, inner wall of cavernous sinus and floor of sellaturcica as well as tumor’s biological behavior. If a Intrasellar stalk CP grows into a giant tumor in vertical direction, grading the relationship between tumor and hypothalamus is the same as that of Suprasellar stalk CPs.

Since Central type CP grows within and along pituitary stalk, we are unable to identify its definite origin site or tumor base. Considering hypothalamus damage can always be found in each Central type case, we speculate it would be the same origin as Hypothalamic stalk CP, but grows within rather outside the stalk. Combined these two types, CPs with hypothalamic origination are account for 67.4% of all CPs. In other words, only 32.6% of CPs, at most, may not involve hypothalamus and can be safely radically resected with less or no hypothalamic damage.

### Growth pattern

**Figure 11:**
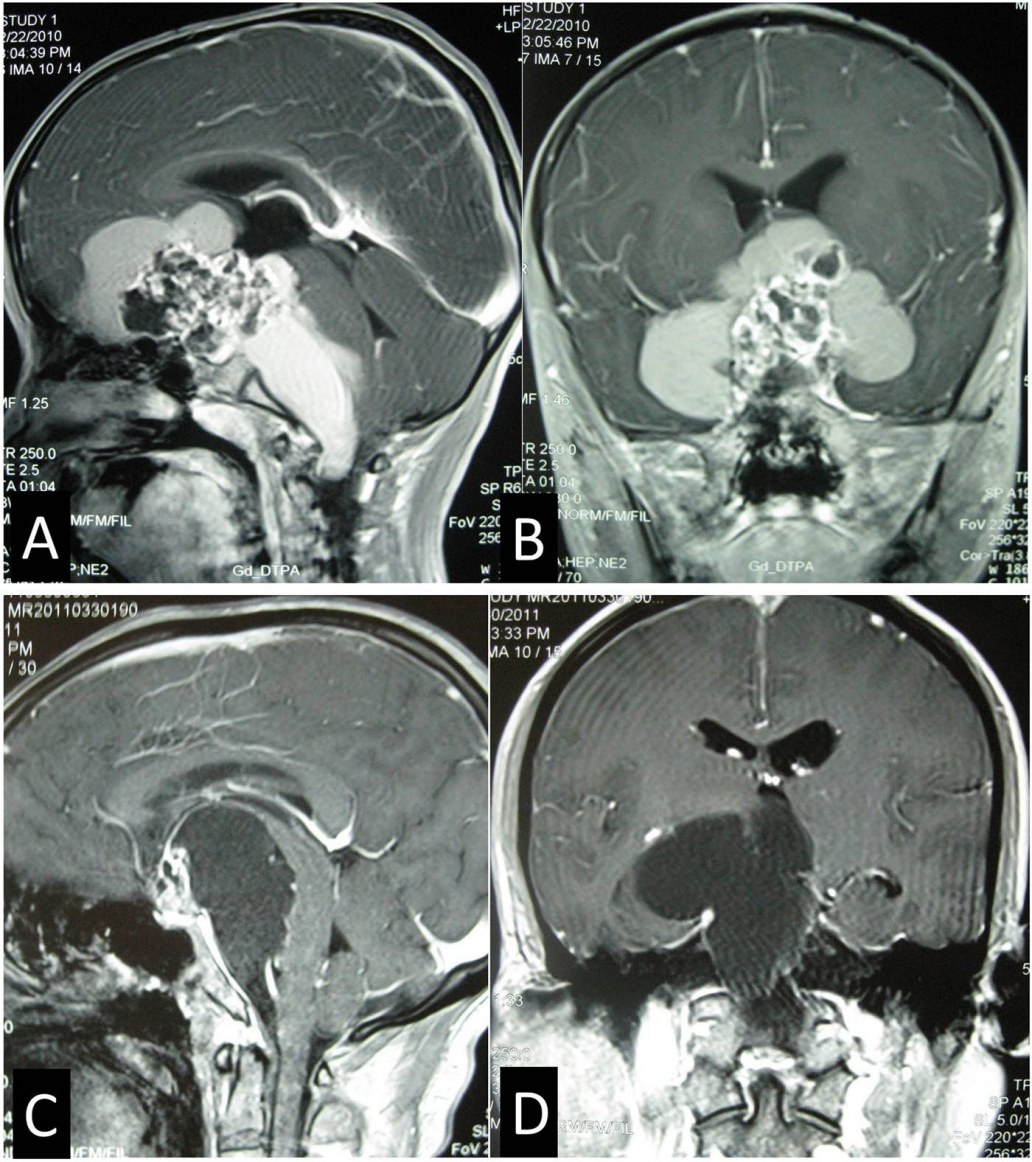
Examples of giant Suprasellar stalk CPs grow in roughly horizontal direction. (**A** and **B**) Sagittal (A) and Coronal (B) postcontrast T1-weighted MR images showing a giant Suprasellar stalk CP extends to the anterior fossa, sylvian fissure, the prepontine and posterior fossa. (**C** and **D**) Sagittal (C) and Coronal (D) postcontrast T1-weighted MR images showing a giant Suprasellar stalk CP extends to the prepontine and posterior fossa.

**Figure 12:**
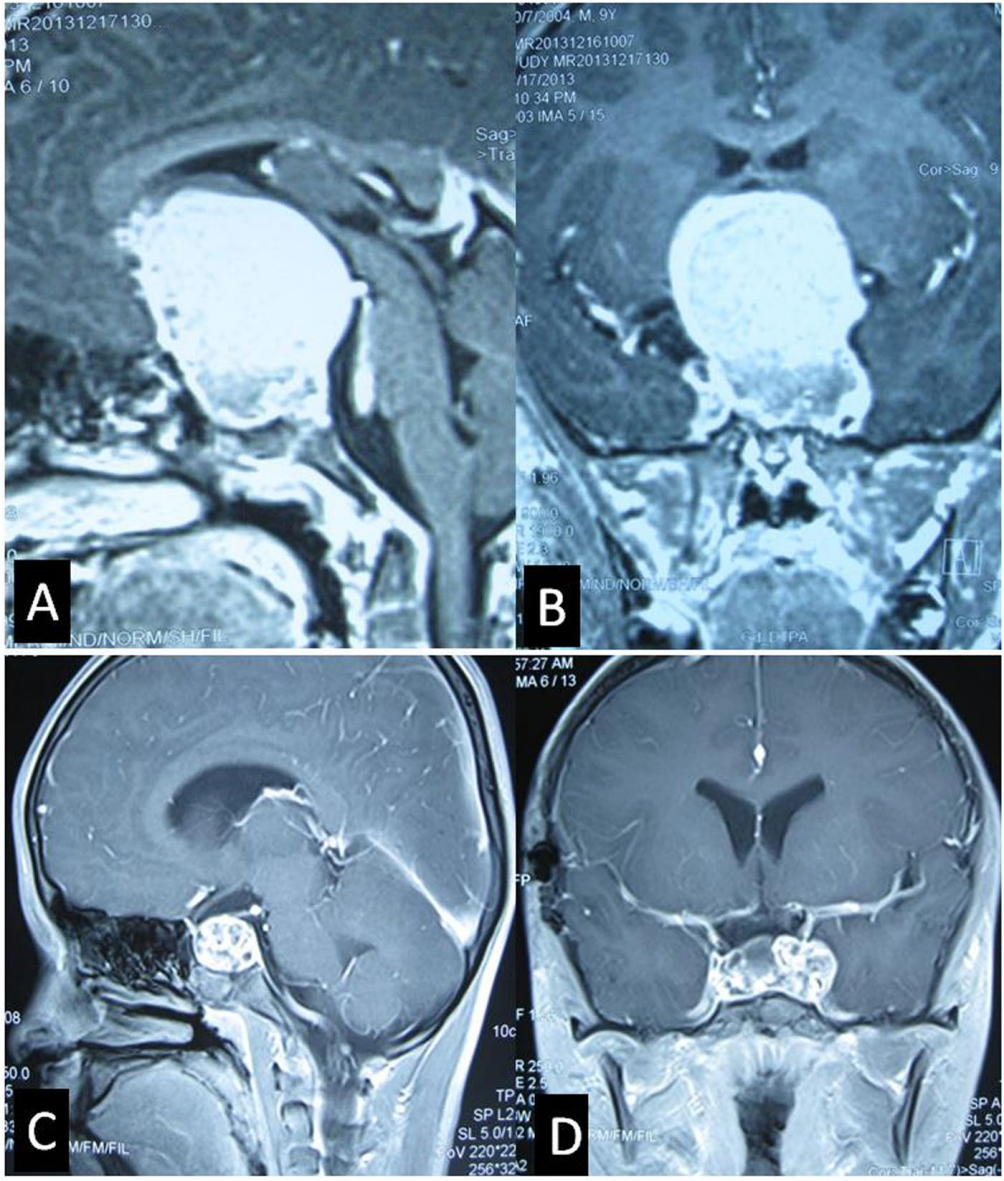
Growth pattern of Intrasellar stalk CPs. (**A** and **B**) Sagittal (A) and Coronal (B) postcontrast T1-weighted MR images showing a giant solid Intrasellar stalk CP grows in vertical direction and pushes the hypothalamus upward, at the same time, sella turcica is obviously much enlarged by the tumor. (**C** and **D**) Sagittal (C) and Coronal (D) postcontrast T1-weighted MR images showing a Intrasellar stalk CP grows laterally into the left cavernous sinus.

Each subtype with different origin basically presents different growth pattern, that is growing in vertical or in horizontal direction (Fig. 6). Generally hypothalamus- originated CPs, including Central type and Hypothalamic stalk type, commonly expand more in height than in breadth, extending into the third ventricle, lateral ventricle and even interhemispheric fissure, or transinfundibularly down into the intrasellar cavity. This growth feature is more obvious in Central type (Fig. 4) than in Hypothalamic stalk type (Fig. 9). However, the growth pattern of Suprasellar stalk CPs (non hypothalamus-originated) is just the opposite. They tend to expand more in breadth than in height, extending along the space of skull base cisterns to the parasellar region, prepontine and posterior fossa, anterior fossa and sylvian fissure, and in some cases to be a giant tumor (Fig. 11). Intrasellar stalk CPs grow more like a pituitary adenoma, expanding more vertically to elevate the third ventricle floor except the diaphragm is penetrated, as well as laterally into cavernous sinus and downward into sphenoidal sinus (Fig. 12).

### Infiltration and adherence

In the medical literatures, terms like “adherence”, “involvement”, “invasion”, etc have been frequently used to describe the attachment of CPs to the contiguous brain structures such as hypothalamus, optic chiasm, brain tissue, artery and stalk^19,32,41–43^. However, these terms are imprecise and subjective, and mostly depend on the neurosurgeon’s personal interpretation. More importantly, the definitions did not reflect the intrinsic difference of the relationship of the origin area from that of other non-origin surface of the tumor with the neighboring structures. Although a few of studies on the boundary between CP and surrounding tissue have been reported^44^, the distinction of pathological changes between origin place and other non-origin place has not been investigated.

CP is well known as a well- circumscribed, encapsuled mass. However, it has been reported in the autopsy studies that in some area between the tumor and the surrounding brain parenchyma, an indistinct demarcation was observed. Under the wider and direct visualization of endoscopy, we also observed that at the origin place, the cleavage plane of the tumor is disappeared and the infusion occurs between tumor and hypothalamus and/or pituitary stalk. Based on the surgical and autopsy observation, we hypothesized that an infiltration of CP to the adjacent structure only occurs at the origin site, while an expanding compression occurs at the non-origin surface of CP. In other words, only the origin site of hypothalamus and/or stalk would be infiltrated by CPs, the other surrounding structures such as optic chiasm, blood vessels, other brain tissue, and “non-origin” hypothalamus and stalk is just pushed and compressed by expanding CPs, resulting in a loose or dense adherence.

In order to confirm our hypothesis, the surgical specimens of the origin and non-origin capsule of CPs were pathologically studied respectively in 8 cases (including 4 cases of Hypothalamic stalk CPs, 3 cases of Suprasellar stalk CPs and 1 case of Intrasellar stalk CPs) of admantinomatous variants. The results demonstrated that the typical interdigitation or finger-like infiltration protruding into the hypothalamus was observed in the origin tissue specimens in Hypothalamic stalk CPs (Fig. 5A1-3). But in the specimens of tight adherence area of hypothalamus in Suprasellar stalk CPs, this typical histopathological feature was not observed, instead of a relative smooth surface composed of the palisaded tumor cells in some cases (Fig. 5B1-3). In Hypothalamic stalk CPs and Suprasellar stalk CPs, the stalk where the tumor originated from was full of tumor cell infiltration interspersed with the remnants of stalk structure (Fig. 5C1-3). Although extensively adhering to surrounding structure such as optic chiasm and basal ganglion, the other non-origin capsule showed a relative smooth and clear, or irregular interface without any interdigitated infiltration (Fig. 5D1-3).

Therefore, we propose a new concept to describe and understand the relationship between the interface of CPs and the surrounding structure, that is, invasion or infiltration for origin site, adherence for non-origin surface. In our operative observation, the extent of infiltration at the origin site can be broad or narrow. In the broad cases of Hypothalamic stalk CPs, both the tuberal area and the lateral part of hypothalamus can be involved, total tumor removal often leads to a massive defect in the third ventricle floor, with only sparing mammillary region. In the narrow cases, the infiltrating extent is often confined to the tuberal area of hypothalamus, the lateral part of hypothalamus is compressed but still exists and become thicker and thicker toward lateral side. After tumor removal, considerable hypothalamic tissue can be preserved with a little defect of the third ventricle floor. Therefore, sharp dissection under direct visualization is the paramount to preserve hypothalamus function during surgery. The adherence of the non-origin surface to tumor can be loose or tight, probably depends on the degree of chronic inflammatory reaction^44–46^. Focusing on the tumor- hypothalamus interface, which is of fundamental importance to the surgeon, there are two distinct relationships between tumor and hypothalamus: infiltration in Hypothalamic CPs and adherence in Suprasellar stalk CPs and Intrasellar stalk CPs if the hypothalamus is involved.

### Hypothalamus damage

Preserving the hypothalamic function is critical in the surgical management of CP, particularly in an aggressive resection against the risk of significant morbidity and poor long term outcome of quality of life. With the aid of pre- and post-operative imaging studies, several reports have proposed their grading systems to define and score the hypothalamic involvement in CP patients. However, the study of detailed extent of the hypothalamic damage based on direct operative observation under endoscopy is missing in the literature.

**Figure 13:**
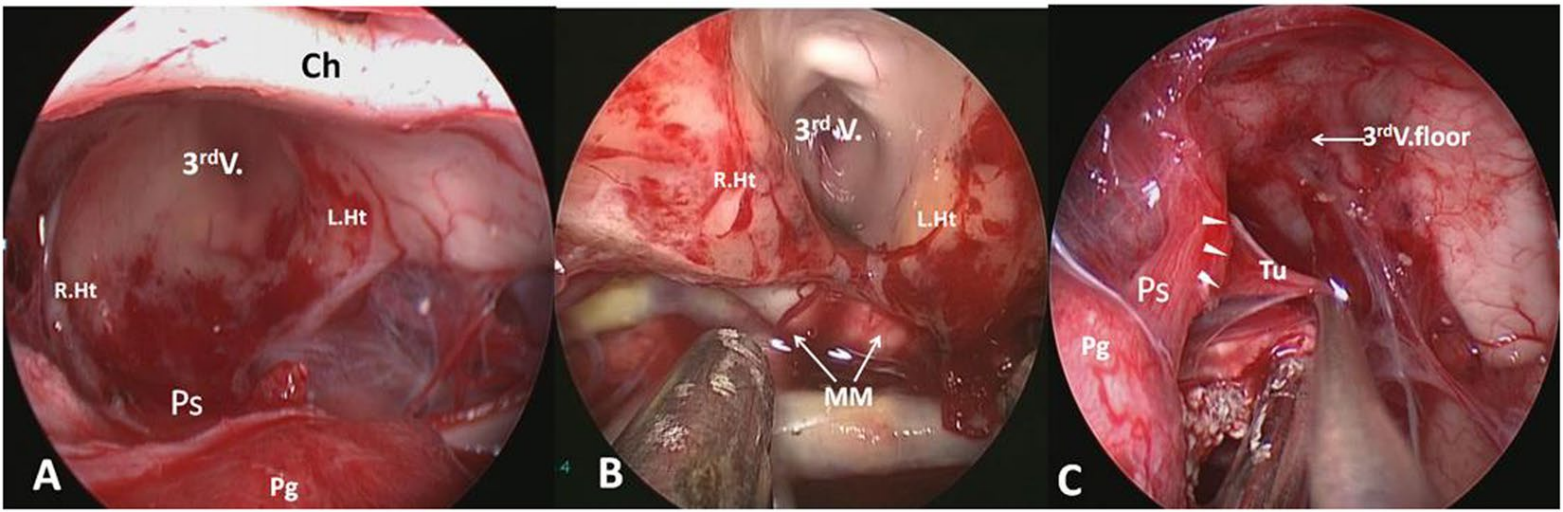
Different subtype of CPs presents different relation pattern to hypothalamus. (**A**) Ipsilateral hypothalamus injury (left): after removal of a Hypothalamic stalk CP, a defect at the left side of the third ventricle floor could be seen, the remnant of stalk was pushed to the right side and connected to the right side of the third ventricle floor, with no hypothalamus damage at the right side. (**B**) Bilateral hypothalamus injury: after removal of a Central type CP, a wide defect of bilateral floor of the third ventricle was observed, making the third ventricle totally opened with no or a small area of remnant third ventricle floor and stalk. (**C**) Removal of a Suprasellar stalk CP which originated from the lower-middle portion of stalk (white arrowheads), pituitary stalk was pushed to the right side, and the third ventricle floor was intact, with no hypothalamus damage. Tu, tumor; Pg, pituitary gland; Ps, pituitary stalk; 3^rd^ V., the third ventricle, L.Ht, left hypothalamus, R.Ht, right hypothalamus; MM, mammillary body.

Different subtype of CPs presents totally different relation pattern to hypothalamus (Fig. 6). Radical resection of Hypothalamic stalk CPs must cause hypothalamus damage in varying degree due to their origination. Two categories of hypothalamic damage have been observed after gross tumor removal: i) ipsilateral injury, tumor often grows toward to one side, or retroinfundibular or preinfundibular side and invades that side of hypothalamus. Completely surgical removal can cause a defect at the floor of the third ventricle. The area of absent floor is depended on the extent of tumor infiltration, but mostly limited to ipsilateral side, or anterior or posterior half of hypothalamus, depending on the position of stalk. The remnant of stalk connects to nearly intact opposite side of the floor of the third ventricle, and all of these structures are compressed and pushed to contralateral side, away from the midline (Fig. 13A); ii) bilateral injury, after completely surgical removal of lesion, a wide defect of bilateral floor of the third ventricle is observed, making the third ventricle totally opened with no or a small area of remnant floor of the third ventricle and stalk (Fig. 13B). The extent of the defect in both categories is depending on the equator of the tumor between cisternal part and intraventricle part. Given that the defect exists in the area of the tuber cinereum even in a small size of CP and that more lateral more thicker the remnant hypothalamus become, we believe that the tumors first originate or invade the area of the tuber cinereum and gradually enlarge and expand toward to the lateral area of hypothalamus in one side in ipsilateral type, and in both side in bilateral type. The ipsilateral type represents the most common category, accounting for 83.3% (30/36) of Hypothalamic stalk CPs. It has been reported that unilateral hypothalamus damage can hardly cause complete loss of hypothalamus function owing to contralateral compensation^47^. Therefore, it is important to preserve a contralateral remnant of hypothalamus during surgery. In the contrary, if the defect in the bilateral type is massive, the postoperative complication is much more severe with higher morbidity and mortality and poor long-term follow up outcomes.

Resection of Central type CPs also leads to bilateral hypothalamus injury, but typically limited to bilateral tuber cinereum if the tumor is smaller. Occasionally, if the tumor is larger, the injury can extend to the entire hypothalamus (Fig. 13B).

Removing Suprasellar and Intrasellar stalk CPs causes less or no hypothalamic damage due to their non hypothalamic origin (Fig. 13C). The tumor can elevate and compress the floor of the third ventricle in Grade 1 and Grade 2 patients. Carefully dissection of the tumor capsule under direct view can keep the floor nearly intact or less injury. Only in Grade 3 patient, hypothalamic damage could be obvious because the tumor expands into the third ventricle.

### The novelty of our classification

Our new classification scheme is not to deny but rather supplement previous classifications with more emphasizing on the origin of the tumors. The novelty of our scheme and difference with other endoscopic classification are briefly listed as follows:

1. With our classification, we can identified what is the exact subtype of Peripheral or Central type in most patients, totally independent of the recognition of the pituitary stalk, which is difficult to identified in preoperative MRI, and without the need to classify pre- or retro infundibular type, with which, it is hardly to classify the tumor with the pituitary stalk located to one side (right or left).
2. The origin of the tumor, which is critical to determine the exact relationship of tumor to hypothalamus and the extent of resection, has not been considered according to Kassam’s classification. In contrast, only do Hypothalamic stalk CP and Central type origin from and invade hypothalamus, while other types have no relationship to or compress but not invade hypothalamus in our classification scheme.
3. Currently, the terms like ‘adherence’, ‘invasion’, ‘involvement’ etc used to describe the relationship between tumor and adjacent structures is confusing. We first provided the evidence that where is infiltrated, where is not by tumor, making the relationship between tumor and surrounding structures more clear and distinct. The significance of this concept is obvious. Beside of correctly understanding the exact relationship among neighboring structures, particularly with hypothalamus, we can also use the term precisely to discribe the relationship: infiltration or invasion for origin place, adherence for non-origin capsule. For example, for the relation with optic chiasm, it is inappropriate to use the term like invasion, but adherence although it can be tight or loose. For hypothalamus and stalk, either invasion or adherence can be used depending on the tumor originated from them (infiltration or invasion) or not (adherence).
4. With our classification, we can predict the extent of hypothalamic injury before aggressive resection, and therefore determine operative strategy. The intraoperative hypothalamus injury is not depend on tumor size, but rather depend on the origin and its extension: broad or narrow. A large Suprasellar or Intrasellar stalk CP may have no or little hypothalamic damage, while a small Central type or Hypothalamic stalk CP can cause severe hypothalamic damage after radical resection if the infiltration of the origin is extensive.
5. We provided a percentage proportion data for each subtype. We first reported that nearly 70% of CPs (including Central type and Hypothalamic Stalk type) originated from hypothalamus. The radiological characteristics and growth pattern of each subtype was first described. Therefore, each subtype can be identified on the preoperative MRI image study in most of cases with CPs.
6. We first proposed a grading system to evaluate the compression degree to hypothalamus in the patients with non hypothalamic-origin tumors (including Intrasellar and Suprasellar stalk CPs). In addition, the differentiation between Intrasellar stalk CP with extending to suprasellar space and Suprasellar stalk CP with extending to intrasellar cavity was also described, which is seldom reported in the literature.
7. Our classification is more systemic, comprehensive and practical, rather than fragment, indistinct and even incorrect. We can roughly estimate the subtype of CP in most of patients from preoperative image study based on the radiological characteristics, growth pattern of each subtype and age, and then predict the possible extent of hypothalamic injury, and finally determine the operative strategy: radical or limited resection.

In conclusion, a good infrachiasmatic exposure and high definition wide-angle visualization of endoscopic endonasal approach allow surgeon clearly identifying the stalk, hypothalamus, origin site and other relation of CP to surrounding structures during the procedures for resection of lesion. Knowing the origin of CP in individual case is decisive to understand its growth pattern, relation to vital structures, preserving the hypothalamic and neuroendocrine functions. Based on the relation to pituitary stalk, CPs can be divided into two types: Peripheral and Central types. Peripheral type is further subclassified into three categories: Hypothalamic, Suprasellar and Intrasellar stalk CPs. Each subtype has totally different growth pattern and different relationship with hypothalamus and stalk. Understanding these differences of CPs is essential prior to planning the most appropriate surgical approach and degree of excision, and predicting the postoperative morbidity and prognosis of the patients.
